# Cryo-EM structure of the human Kir7.1 channel reveals the molecular basis of snowflake vitreoretinal degeneration disease

**DOI:** 10.1038/s42003-026-10436-0

**Published:** 2026-06-21

**Authors:** Niamh O’Malley, Chady Nasrallah, Ashton Churchill, Jay Bertrand, Belinda R. Faust, B. A. Wallace

**Affiliations:** 1 Institute of Structural and Molecular Biology, Birkbeck University of London, Malet Street, London, UK; 2 Vertex Pharmaceuticals, 86-88 Jubilee Ave, Milton, Abingdon, UK; 3 Present Address: Evotec US, 303B College Rd E, Princeton, NJ USA; 4 Present Address: The Francis Crick Institute, 1 Midland Road, London, UK

**Keywords:** Cryoelectron microscopy, Potassium channels

## Abstract

The inward rectifying human potassium channel 7.1 (Kir7.1) is a vital ion channel involved in maintaining cellular homoeostasis and electrical signalling across various tissues and organs, activated by phosphatidylinositol 4,5-bisphosphate (PIP_2_). A genetically inherited loss-of-function mutation in Kir7.1 (R162W) has been linked to the rare retinal disease Snowflake Vitreoretinal Degeneration (SVD), for which there are currently no curative treatment options. Here, the cryo-EM structures of wild type Kir7.1 and the R162W disease-related variant are presented, which unveil the molecular basis of SVD: the reorientation of the mutant tryptophan side chains into the pore impedes the flow of potassium ions, which would result in the loss of Kir7.1 transport function. Furthermore, this investigation shows that PIP_2_ binding widens the helix bundle crossing gate diameter, even in the absence of a docked cytoplasmic domain. This observation contrasts with other Kir-PIP₂ complexes and suggests that Kir7.1 may adopt an intermediate conformation during channel activation. These findings provide a structural basis for Kir7.1 loss of function in SVD and provide a framework for future therapeutic development.

## Abstract (web summary)

Editor Summary: Cryo-EM structures of Kir7.1 suggest the SVD-associated R162W mutation may block the pore and impair ion transport, offering a structural basis for disease

## Introduction

Inwardly rectifying K⁺ (Kir) channels are essential regulators of membrane potential and cellular excitability across diverse tissues. Dysfunction of these channels, known as channelopathies, can have severe pathological consequences. Kir7.1, the most recently identified member of the Kir channel family, differs from classical Kir channels by its unusually low single-channel conductance. It is widely expressed throughout the human body, notably in the retinal pigment epithelium (RPE), trachea, uterine smooth muscle, small intestine, and choroid plexus^1^. The channel is vital for viability as Kir7.1-deficient mice die shortly after birth^2^.

Mutations in the human *KCNJ13* gene, which encodes Kir7.1, are associated with two retinal diseases: Snowflake Vitreoretinal Degeneration (SVD; MIM #193230), and Leber Congenital Amaurosis (LCA; MIM #204000)^3–9^. SVD is characterised by snowflake-like retinal deposits, fibrillar vitreous degeneration, retinal detachment, and early-onset cataracts. In the eye, Kir7.1 is localised to the apical processes of the RPE, where it co-localises with the Na^+^/K^+^ pump and plays a critical role in maintaining ion homoeostasis in the subretinal space, which is essential for photoreceptor health^10^. The RPE and photoreceptors share a tight sub-retinal space where, upon light exposure, K^+^ concentration can drop from 5 to 2 mM. Kir7.1 facilitates K^+^ efflux from RPE cells into the subretinal space to compensate for this decrease, and within the RPE, Kir7.1 contributes to a normal electroretinogram, with shRNA silencing resulting in c-wave amplitude reduction^11^.

A single-point mutation in Kir7.1, substituting arginine 162 with tryptophan (R162W), is responsible for SVD through a loss of channel function^12^. This mutation impairs Kir7.1’s ability to conduct potassium ions and maintain ion homoeostasis in the subretinal space^13^. Despite its clear clinical impact, the precise molecular mechanism by which R162W impairs Kir7.1 activity and contributes to retinal degeneration remains incompletely understood.

Eukaryotic Kir channels are modulated by different ligands depending on their physiological role; however, all human Kir channels require phosphatidylinositol 4,5-bisphosphate (PIP_2_) for activation^14^. In isolated bovine RPE cells, Kir7.1 channel activity is tightly regulated by the local availability of PIP_2_^15^. Crystallographic studies of related Kir channels have identified the PIP_2_ binding site at the interface between the transmembrane domain (TMD) and the cytoplasmic domain (CTD)^16^. Nonetheless, the detailed mechanism of PIP_2_-induced channel activation remains unclear, particularly for the less extensively studied Kir7.1 channel.

This study presents the cryo-EM structures of Kir7.1 in both wild type (WT) and SVD variant forms. The structures reveal that the R162W mutations disrupts ion conduction by positioning bulky tryptophan side chains within the channel pore, effectively blocking ion flow. These findings offer molecular insights into the pathogenic mechanism underlying this rare retinal disorder. Furthermore, the PIP_2_-bound structure described herein provides insight into Kir7.1-specific regulation by this lipid.

## Results

### The overall structure of Kir7.1

**Fig. 1:**
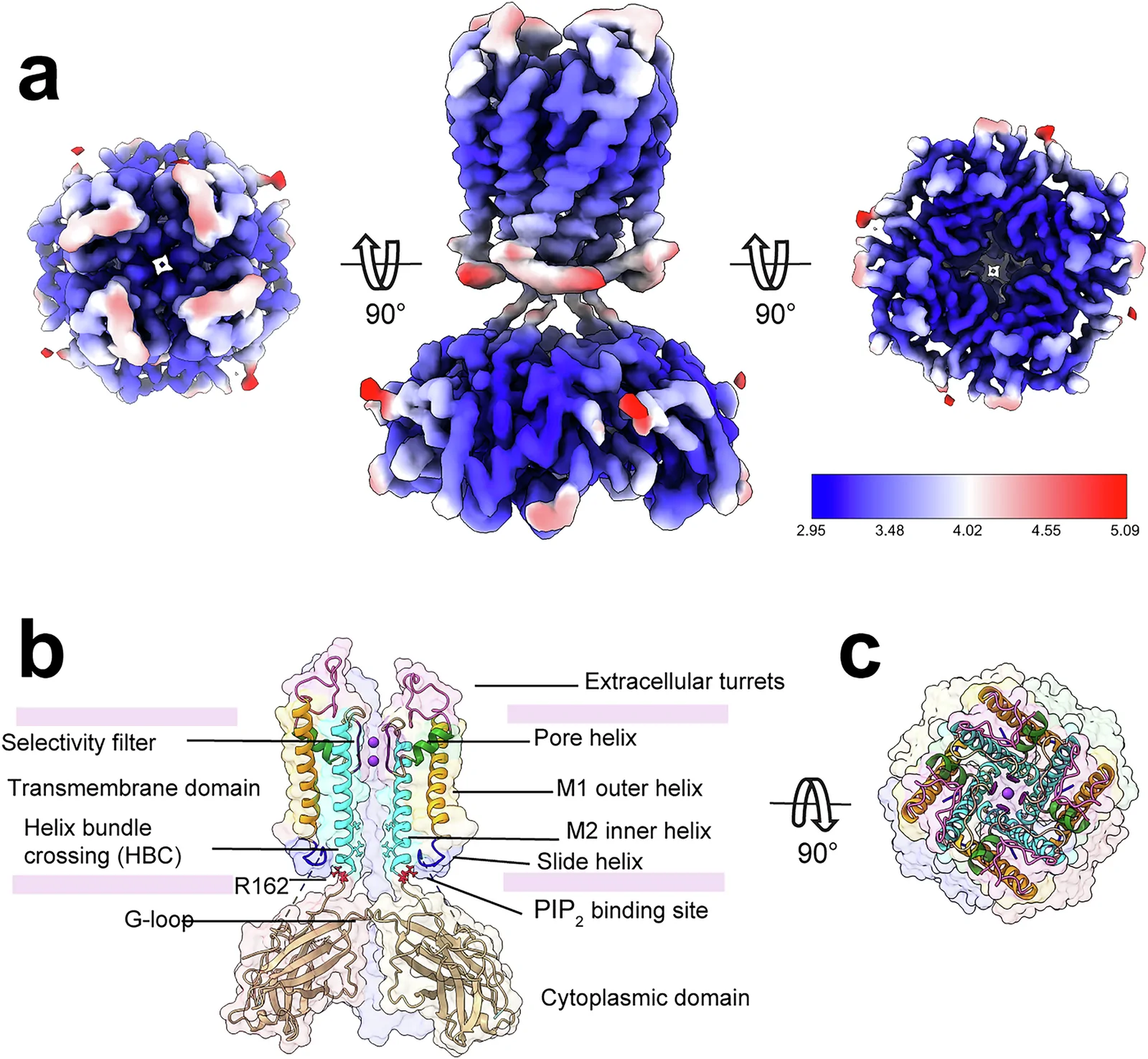
Structure of the human Kir7.1 channel. **a** Cryo-EM maps of Kir7.1 at 3.54 Å resolution showing cytoplasmic (left), lateral (middle) and extracellular (right) views. The map is coloured based on local resolution estimates ranging from 2.95 to 5 Å, with the corresponding colour scale bar shown below the maps (Å). **b** Schematic (ribbon) diagram of two subunits of the atomic structure of Kir7.1 overlaid on the electron density of the structure. Three subunits are shown in transparent colour surface. Key Kir channel features are coloured and labelled: outer helix (M1, yellow), inner helix (M2, cyan), pore helix (green), slide helix (blue), selectivity filter (purple), extracellular turrets (pink), g-loop, helix bundle crossing (HBC), arginine 162 (R162) and PIP_2_ binding site. Pink bars represent the plasma membrane. **c** Top view of the Kir7.1 tetrameric structure from the extracellular side. The transmembrane domain is displayed in ribbons and coloured according to the structural elements described. The cytoplasmic domain is shown as transparent surface. Potassium ions residing within the selectivity filter are coloured purple.

Tagged full-length Kir7.1 was recombinantly expressed in Expi293F cells using transient transfection, and purified for structural analysis. Size exclusion chromatography indicated that the protein formed a homotetrameric complex. Cryo-EM analysis of the full-length human Kir7.1 channel determined the structure at 3.54 Å resolution revealing a tetrameric quaternary structure (Fig. 1a). Weak density protruding from residue N95, a known glycosylation site in the extracellular turret, suggests the presence of a mature, glycosylated form of the ion channel (Supplementary Fig. 1). The reconstruction displayed structural elements typical of Kir channels: in all 4 subunits, two alpha helices span the membrane (M1 and M2), while the third, smaller pore helix points inwards, forming the selectivity filter (Fig. 1b, c). The TMD is connected to the CTD by a flexible TMD-CTD linker that was less resolved due to its intrinsic flexibility. Density was resolved for two potassium ions within the selectivity filter. 3D variability analysis demonstrated swinging and rotation of the CTD in relation to the TMD facilitated by this disordered linker region.

### Kir7.1 in complex with PIP_2_

**Fig. 2:**
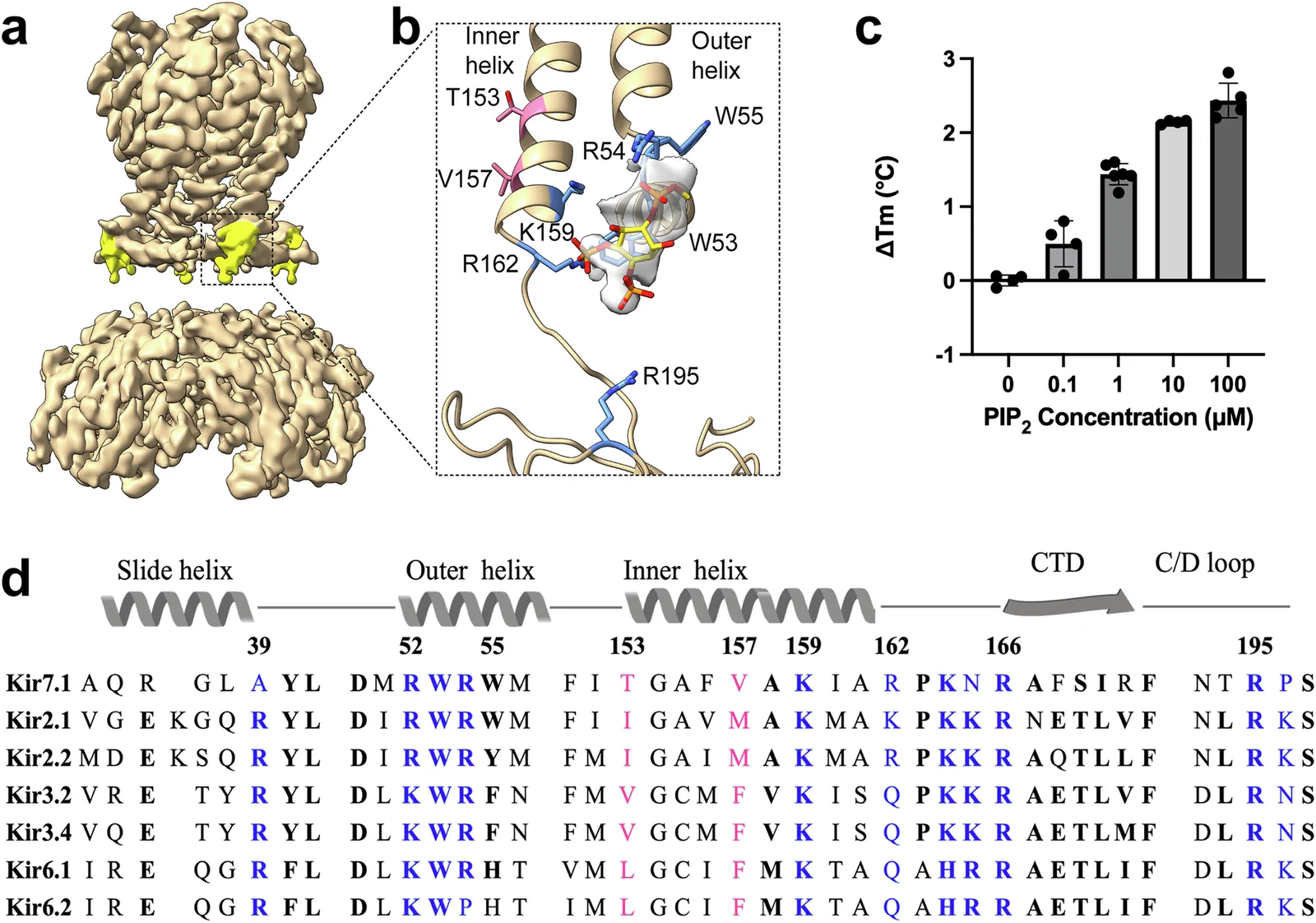
The Kir7.1-PIP_2_ complex. **a** Cryo-EM structure of the Kir7.1 tetramer with four PIP_2_ molecules bound. Kir7.1-PIP_2_ EM density map is shown with density relating to the PIP_2_ ligands coloured in yellow. **b** Enlarged view of the PIP_2_ binding pocket with PIP_2_ densities shown in grey mesh visualised at a contour threshold of 0.14. **c** Thermal stability of Kir7.1 in response to increasing concentrations of PIP_2_ measured by NanoDSF. ΔTm values were calculated relative to apo-Kir7.1. Bars show the mean ± standard deviation (*n* = 4-5 technical replicates). **d** Amino acid sequence alignment of eukaryotic Kir channels. Residues previously identified in other Kir channels as interacting with PIP_2_ are highlighted in blue. Residues located in the helix bundle crossing are coloured pink. Residues are numbered according to Kir7.1 sequence.

Kir7.1, like all human Kir channels, requires PIP_2_ for channel activity^17^. Direct activation of Kir7.1 by PIP_2_ has been demonstrated through electrophysiology experiments^15,17^. To investigate the structural basis of this activation, we determined a cryo-EM structure of Kir7.1 in complex with a short-chain (dioctanoyl) PIP_2_ at 3.29 Å resolution. PIP_2_ was bound in the canonical binding site observed in other Kir channels^18,19^, where it interacts with residues W53 and R54 in the outer helix (Fig. 2a, b). No direct interaction was observed between PIP_2_ and residues within the flexible TMD-CTD linker or the cytoplasmic domain. Nano differential scanning fluorometry (NanoDSF) was used to assess the thermal stability of Kir7.1 in the presence of PIP_2_. It was found to stabilise Kir7.1 in a dose-dependent manner (Fig. 2c).

### PIP_2_ binding widens the helix bundle crossing in Kir7.1

**Fig. 3:**
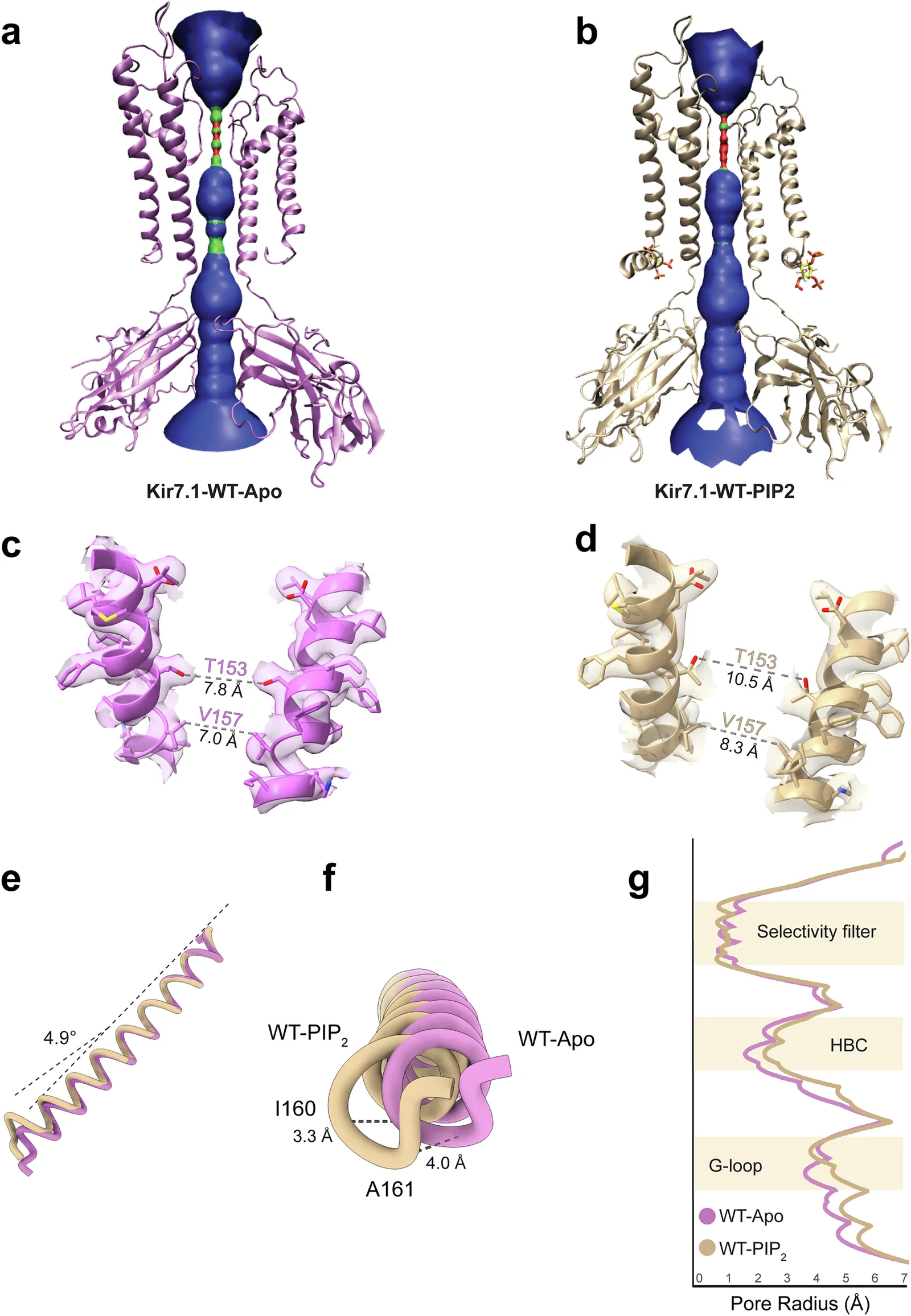
Opening of the helix bundle crossing. Top: Plot showing the accessible interior of the pore calculated using HOLE2 software of **a** apo Kir7.1 and **b** Kir7.1-PIP_2_, where blue surfaces are pore radius >2.3 Å, green surfaces are radii between 1.2 and 2.3 Å, and red <1.2 Å^36^. Middle: Lateral view of the HBC for **c** apo Kir7.1 and for **d** Kir7.1-PIP_2_ with EM density showing backbone and side chain fit. Distances were measured from the hydroxyl oxygens of T153 and the side chain methyl groups of V157. Measurements were calculated in ChimeraX. Bottom: **e** 4.9° bending of the M2 inner helix in the PIP_2_ model (tan) compared to that in the apo model (pink). **f** View from the cytoplasmic side of the M2 helices showing a 3.3 Å difference between the Cα atoms of residue I160 and a 4.0 Å difference between the Cα atoms of residue A161 in the apo (pink) and PIP₂-bound (tan) structures. **g** Plot of pore radius (between van der Waals surfaces) calculated using HOLE2 along the membrane perpendicular for the apo (pink) and PIP_2_ (tan) structures.

The helix bundle crossing (HBC), located at the base of the inner helices, is a key gating region in Kir channels^20–22^. In the PIP_2_-bound Kir7.1 structure, the pore at the HBC was wider than in the apo Kir7.1 model (Fig. 3). This dilation resulted from a slight bending of the inner M2 helices, bringing the bottom of the helices closer to the PIP_2_ binding site. This resulted in two key residues, T153 and V157 being positioned further apart (Fig. 3c, d). The M2 helix hinged at glycine 154, bending by 4.9°, which displaces the end of the helix by ~3.5 Å compared to the apo model (Fig. 3e, f). G154 is conserved among Kir channels and is known to be required for the bending of the inner helices and a similar widening of the HBC was observed in Kir2.2 and Kir3.2 docked structures (Supplementary Fig. 2)^21^. The G-loop gate, another region commonly constricted in Kir channels, remained dilated and open in both the apo and PIP_2_-bound states of Kir7.1.

### Kir7.1-R162W mutant causes Snowflake Vitreoretinal Degeneration by blocking ion conduction pathway

**Fig. 4:**
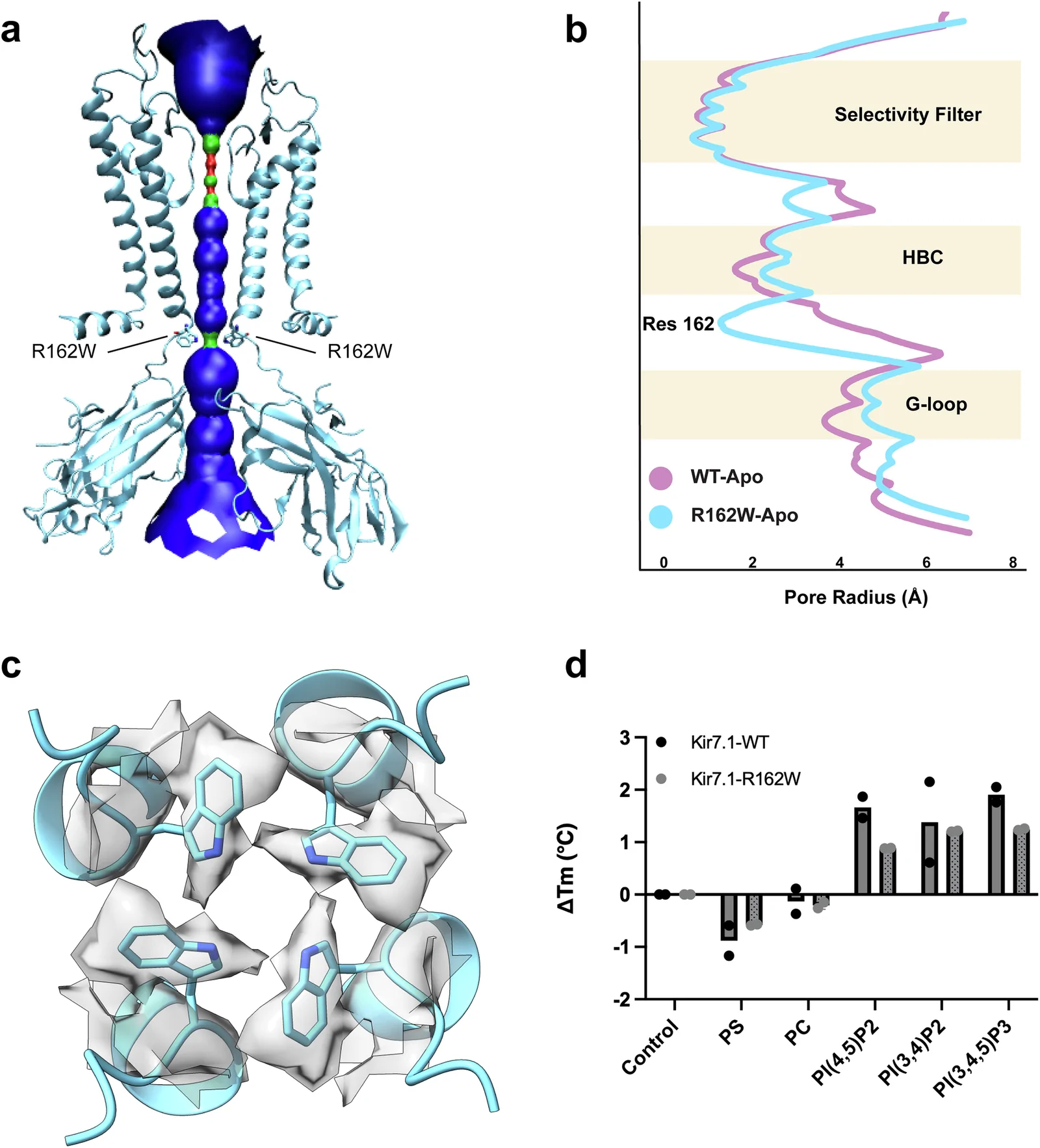
R162W mutation blocks channel pore of the Kir7.1 channel. **a** Plot showing the accessible interior of the Kir7.1-R162W pore calculated using HOLE2 software^36^. Tryptophan 162 in the TMD-CTD linker region is shown. Blue surfaces are where the pore radius is >2.3 Å, green surfaces are for regions where the pore radius is between 1.2 and 2.3 Å, and red surfaces are where the pore radius is <1.2 Å. **b** Plot of the pore radius (between van der Waals surfaces, calculated with HOLE2) for the WT apo (pink) and R162W (blue) structures. **c** View from the intracellular side of residue 162 in Kir7.1-R162W showing the tryptophan side chains within the electron density (3.96 σ contour). **d** NanoDSF experiment measuring change in Tm of Kir7.1-WT and Kir7.1-R162W in the presence of lipids. Lipids were added at a final concentration of 25 μM. Control measurements contained protein only. WT is shown in solid colour and R162W mutant in spots. An increase in Tm was observed for the PIPs but not for PS and PC. Two biological replicates were performed, each including three technical replicates per condition. Data are represented as mean values of biological replicates.

Kir7.1-R162W was expressed and purified from Expi293F cells, and its size exclusion chromatography elution profile was similar to what was observed for the WT protein, indicative of a folded tetrameric structure (Supplementary Fig. 3a). The comparable protein yield and thermal stability between the mutant and wild-type proteins suggested that the mutation does not significantly compromise protein stability (Supplementary Fig. 3b). The cryo-EM structure of the Kir7.1-R162W mutant was determined at 3.5 Å resolution. The overall architecture of the mutant channel was comparable to the WT structure, with a global root mean square deviation (r.m.s.d.) of 1.6 Å for all atoms. However, the mutation introduced a notable difference in the TMD-CTD linker region, where clear electron density for tryptophan side chains was observed at the mutation site (Fig. 4c). In the WT structure, this region is less well-resolved, and the arginine side chains are likely dynamic and flexible.

In the Kir7.1-R162W mutant, the bulky tryptophan residues project inward toward the central pore, forming a hydrophobic constriction that reduces the pore diameter from 7 Å to 2.6 Å (Fig. 4b, c). To assess the symmetry of the channel, the cryo-EM dataset was processed using both C4 and C1 symmetry. While the C1 reconstruction yielded a lower overall resolution, clear density for the tryptophan side chains within the pore was still observed (Supplementary Fig. 4).

R162W resides within the PI(4,5)P_2_ binding pocket, and it was of interest to investigate if this mutation would impact the lipid-dependent thermal stabilisation of the protein. We used NanoDSF to measure the thermal stability of the protein in the presence of PIP lipids. Phosphatidylserine (PS) and phosphatidylcholine (PC), which do not interact with Kir7.1, served as negative controls. PI(4,5)P_2_, PI(3,4)P_2_ and PI(3,4,5)P_3_ increased the thermal stability of Kir7.1-R162W, following the same trend as the wild-type, though the effect was reduced (Fig. 4d). Further investigation examined the impact of PI(4,5)P_2_ on the thermal stability of Kir7.1-R162W in a dose-dependent manner, in comparison to the WT (Supplementary Fig. 5). This showed that the R162W mutation reduced the stabilising effect of PI(4,5)P_2_ but did not abolish it. As NanoDSF reports on thermal stabilisation rather than direct lipid binding, these observations may reflect either weakened PIP_2_ interaction or reduced coupling between lipid engagement and conformational stabilisation of the channel.

## Discussion

Kir channels are vital for maintaining potassium homoeostasis across a wide range of tissues, and mutations in these channels are implicated in numerous human diseases^23,24^. One such disease, snowflake vitreoretinal degeneration (SVD), is a rare, debilitating condition currently lacking effective treatment. This study provides structural insight into Kir7.1 dysfunction in SVD, demonstrating that the disease-causing R162W mutation introduces a constriction point in the channel pore that may obstruct ion conduction. Notably, the mutant protein traffics to the membrane and retains a wild-type–like fold, supporting a mechanism of disease rooted in functional blockage rather than protein misfolding.

### R162W structure presents hypothesis for role in SVD

**Fig. 5:**
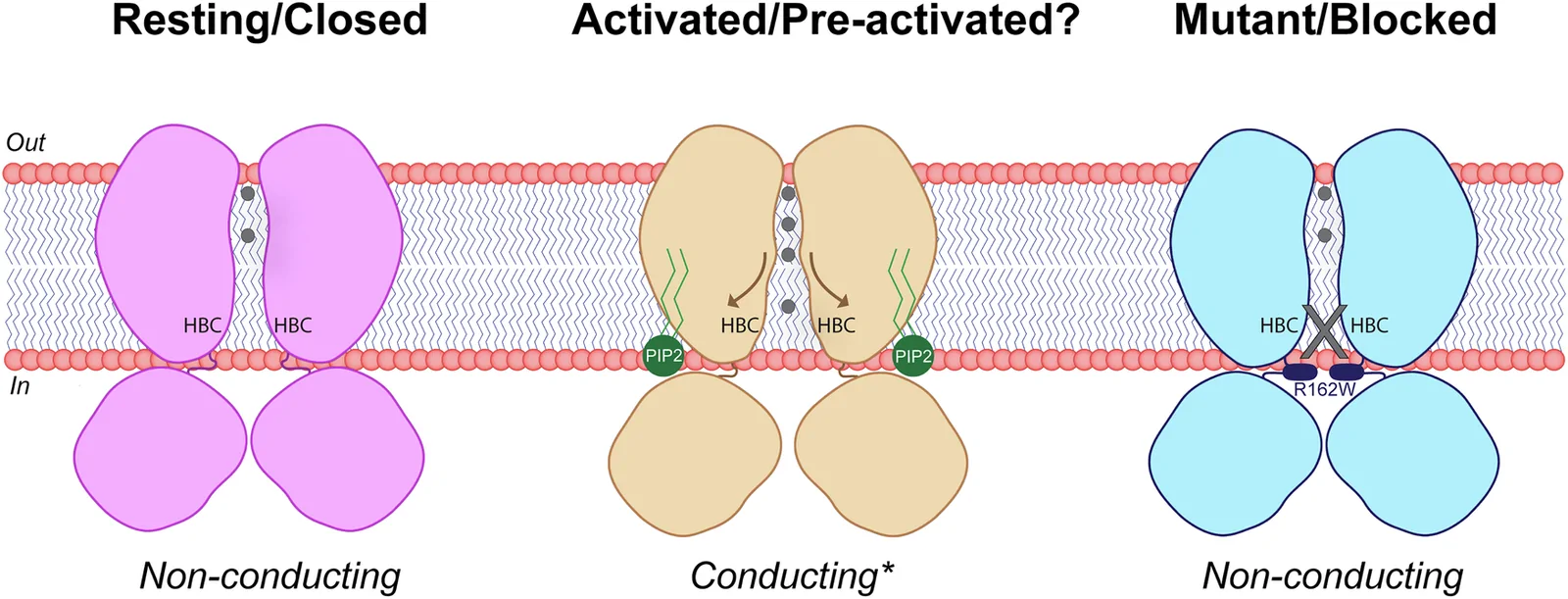
Schematic summary of Kir7.1 activation and pore blockage by R162W mutation. **Left**: Kir7.1 in the resting (closed) state in the absence of the activating lipid PIP_2_. Potassium ions are coordinated by backbone carbonyl oxygens within the selectivity filter (G121, I120 and T119). **Middle**: PIP_2_-bound activated (or pre-activated) state with dilation at the helix bundle crossing. Potassium ions remain coordinated by backbone carbonyl oxygens within the selectivity filter (T122, G121 and I120). Density corresponding to a fourth potassium ion was observed within the central cavity. **Right**: The Snowflake Vitreoretinal Degeneration-associated R162W mutation introduces a steric block within the channel pore, preventing ion conductance. Coordination of potassium ions within the selectivity filter is maintained by backbone carbonyl oxygens (G121, I120 and T119). HBC: Helix bundle crossing. *Note that full activation may require further conformational changes.

Several previous studies have shown that the R162W mutation in Kir7.1 produced a non-functional channel, with injection of mutant Kir7.1 cRNA exhibiting currents indistinguishable from water controls in electrophysiology experiments^13^. Researchers confirmed surface expression and when co-expressed with WT-Kir7.1 R162W-Kir7.1 exerted a dominant negative effect in line with the dominant negative inheritance pattern. Previous hypotheses suggested that the R162W mutation impaired PIP₂ binding by removing a positive charge essential for interaction. This theory arose from the conservation of positively charged side chains within this region in Kir channels and the crystal structure of Kir2.2 bound to PIP_2_ showing a lysine in the corresponding position coordinated to PIP_2_. However, functional studies have shown that smaller, neutral substitutions at this position do not disrupt activity^25^, suggesting that charge is not the critical factor. Instead, our structural findings support an alternative mechanism: the dominant-negative effect of R162W arises from steric obstruction within the channel pore. This is consistent with the inward-facing orientation of the bulky tryptophan side chains, which create a physical constriction (Fig. 5).

Supporting this interpretation, Vera et al. demonstrated through concatemerised Kir7.1 constructs that the presence of just two mutant subunits within a tetrameric channel is sufficient to abolish conductance^25^. Our structural data align with this result - two tryptophan residues projecting into the pore would be enough to introduce a constriction point and prevent ion flow.

Interestingly, a different mutation at the same residue, R162Q, has been linked to retinitis pigmentosa and exhibits a gain-of-function phenotype^8^. A recent structure of Kir7.1-R162Q revealed a docked open conformation even in the absence of PIP_2_, consistent with constitutive channel activation^26^. In contrast, the loss-of-function R162W mutation observed in this study shows the pore occluded by the mutant tryptophan side chains effectively blocking ion conductance. This contrast highlights the role of R162 in regulating Kir7.1 activity and underscores the importance of residue size and side chain chemistry, rather than charge alone, in modulating channel behaviour.

Beyond the disease mutation, this study also provides insight into the gating mechanism of Kir7.1. The channel adopted an open conformation in both the apo and PIP₂-bound states, without transitioning to the fully docked conformation seen in other Kir channels. This suggests that Kir7.1, which is not regulated by G-proteins, may follow an alternative or less tightly coupled activation pathway. PIP₂ binding induced a measurable widening at the HBC, likely facilitated by a bend at the conserved glycine 154 residue. However, the cytoplasmic domain remained in an extended, undocked state, and the G-loop gate appeared constitutively open in all Kir7.1 structures examined.

### Comparison of Kir7.1-PIP_2_ with other activated Kir channels

In contrast to some previously reported Kir channel structures, PIP₂-bound Kir7.1 did not exhibit substantial conformational changes in the cytoplasmic domain (CTD) (Fig. 2a)^18,19^. The channel remained in an ‘extended’ conformation in both apo and PIP₂-bound states. This observation highlights the complexity of Kir channel gating and the potential influence of experimental conditions on structural outcomes.

A variety of structural states have been described across the Kir channel family, with differences in domain arrangement and pore architecture that may reflect the intrinsic flexibility of these channels or context-dependent features of sample preparation. For example, Kir2.2 was originally reported in a ‘docked’ conformation with PIP₂, where the primary conformational change was a widening at the helix bundle crossing, while the G-loop gate remained open (Supplementary Fig. 2)^16^. Kir3 channels have also been shown to adopt docked conformations in the presence of PIP₂, although the degree of conformational change at the G-loop gate has varied across studies, possibly reflecting differences in solubilisation methods, expression systems, or ligand occupancy (Supplementary Fig. 6)^18,27^. Notably, several cryo-EM studies have captured Kir channels in both docked and extended conformations even in the absence of PIP₂, suggesting that the transition between these structural states may be intrinsically dynamic and not strictly dependent on ligand binding^18,28^.

More recently, Kir7.1 structures exhibiting a docked conformation in the presence of PIP₂ have also been reported^26^. These structures were derived from protein expressed in insect cells and solubilised using LMNG with CHS. In contrast, the current study utilised Kir7.1 expressed in human cells and solubilised in GDN, a strategy aimed at preserving features of the native membrane. Expression in mammalian cells can better recapitulate the lipid composition, protein folding machinery, and post-translational modifications of human tissues, as evidenced by the glycosylation of N95 observed in our structure (Supplementary Fig. 1). However, it is difficult to evaluate what influence purification and reconstitution parameters may have on protein structure. It is therefore possible that the extended PIP_2_-bound structure observed here reflects weakened TMD-CTD coupling in GDN, whereas the docked conformation may represent the activated form. Whether the conformation in this study represents a fully activated channel or an intermediate state remains unclear and future work will be needed to determine whether Kir7.1 adopts additional activated states in native membranes or in complex with regulatory cofactors.

Together, our findings suggest that Kir7.1 can adopt a PIP_2_-bound conformation in which the CTD remains extended while the HBC is partially widened. Whether this state represents an intermediate state toward activation or a partially uncoupled conformation is unclear. It is also possible that Kir7.1 requires additional regulatory cofactors or lipids to stabilise CTD docking. There is clearly intrinsic flexibility within Kir channel domains and whether the movement of the CTD and TMD is necessary for channel activation or a feature that happens to be captured structurally remains to be conclusively determined.

In summary, these high-resolution Kir7.1 structures provide key structural and mechanistic insights into the functioning of the channel and its role in disease: (1) the disease SVD may be caused by a blockage of the channel pore by tryptophan side chains; (2) PIP_2_ binds in the predicted site (based on other Kir channel structures) but does not induce CTD docking; and (3) the PIP_2_-bound state displays a widened pore that may represent a previously unobserved pre-activated or intermediate conformation.

## Materials and Methods

### Protein constructs

The full-length human Kir7.1 (UniProt O60928, GENEWIZ) was cloned into a pBcmam plasmid with a FLAG tag at the N-terminus, and its C-terminus was fused with a Tobacco Etch Virus (TEV) cleavage site followed by green fluorescent protein and a 10x-His-tag. The R162W mutation was introduced into Kir7.1 using the Q5 site-directed mutagenesis kit with primers designed using the NEBase changer tool (New England Biolabs). The mutation was confirmed by Sanger sequencing before use (Eurofins, Germany).

### Protein expression and purification

Expi293F (Thermo Fisher Scientific) cells were cultured in Expi293™ expression medium (Thermo Fisher Scientific) in the presence of 8% CO_2_. Cells were transfected with DNA plasmid and ExpiFectamine (Thermo Fisher Scientific) at a density of 3 × 10^6^ cells/ml. After 18 h incubation at 37 °C, the culture was supplemented with enhancer 1 and enhancer 2 (Thermo Fisher Scientific). The cells were collected by centrifugation (500 x *g*, 10 min, 4 °C) after a total incubation time of 48 h and frozen at -80 °C.

Protein was extracted using 1% GDN, 20 mM Tris-HCl, pH 7.5 and 150 mM KCl. The solubilised material was separated by ultracentrifugation at 131,790 x g for 45 min in a 45 Ti rotor (Beckman Coulter). The supernatant was incubated with ANTI-FLAG M2 affinity gel (Sigma Aldrich) and washed with 0.02% GDN, 20 mM Tris-HCl, pH 7.5, and 150 mM KCl. The resin was washed with 2 mM ATP, 20 mM MgCl_2,_ 0.02% GDN, 20 mM Tris-HCl pH 7.5, and 150 mM KCl to remove any protein impurities^29^. The protein was eluted in 0.2 mg/mL FLAG peptide, 0.02% GDN, 20 mM Tris-HCl, pH 7.5, and 150 mM KCl. The purified protein was treated with TEV to remove the GFP-10x-His tag from the C-terminal end. The TEV-cleaved protein was subjected to size-exclusion chromatography purification using a Superose 6 Increase 10/300 GL column (GE Life Sciences) pre-equilibrated with buffer containing 0.02% GDN, 20 mM Tris-HCl, pH 7.5, and 150 mM KCl (Supplementary Fig. 7).

### Grid preparation and cryo-EM data collection

The purified protein was concentrated with Amicon centrifugal filter units with a molecular weight cutoff of 100 kDa, to 7 mg/ml for the wild type protein and 5 mg/ml for the R162W mutant. If not used immediately, the sample was flash-frozen and stored at -80 °C. For the protein-PIP_2_ complex samples, C8-PIP_2_ (Echelon Biosciences) was added to a final concentration of 1 mM and incubated for 30 min. Protein samples were subjected to ultracentrifugation (131,790 x g for 45 min) to remove any aggregates before vitrification. 3.5 µl of sample was applied to a glow-discharged Quantifoil R1.2/1.3 Au 300 mesh grid (Quantifoil), blotted for 4 s with a blotting force of 0 using a Vitrobot Mark IV (FEI) under 4°C and 100% humidity conditions, and then frozen in liquid ethane. The data were collected using a Titan Krios 3Gi microscope (Thermo Fisher Scientific) operated at 300 keV and equipped with a K3 direct electron detector (Gatan) and BioQuantum energy filter (Gatan), operating at 20 eV slit width. A nominal magnification of 130,000 (0.35 Å/pixel calibrated pixel size) was used, with a total dose of 32 e^−^ Å^−2^, fractioned into 50 frames. Cryo-EM data collection statistics are summarised in Table 1.

**Table 1:** Cryo-EM data collection, refinement and validation statistics for Kir7.1 samples

Sample EMDB code PDB code	Apo Kir7.1 (EMDB-55878) (PDB 9TFV)	Kir7.1-PIP_2_ (EMDB-55892) (PDB 9TG6)	Kir7.1-R162W (EMDB-55889) (PDB 9TG3)
**Data collection and processing**			
Magnification	130,000X	130,000X	130,000X
Voltage (kV)	300	300	300
Electron exposure (e–/Å^2^)	31.9	31.9	31.89
Defocus range (μm)	1.2-2.7	1.2-2.7	1.2-2.4
Pixel size (Å)	0.65	0.65	0.65
Symmetry imposed	C4	C4	C4
Initial particle images (no.)	5, 871, 876	8, 821, 241	3, 093, 422
Final particle images (no.)	382, 328	556, 510	140, 275
Map resolution (Å)	3.54	3.29	3.51
FSC threshold	0.143	0.143	0.143
**Refinement**			
Model resolution (Å)	3.54	3.29	3.52
FSC threshold	0.143	0.143	0.143
Map sharpening *B* factor (Å^2^)	-206.6	-194.0	-177.4
Model composition			
Non-hydrogen atoms	8726	9264	8578
Protein residues	1156	1196	1132
Ligands	2	8	2
*B* factors (Å^2^)			
Protein	52.82	82.73	26.06
Ligand	39.45	163.42	24.94
R.m.s. deviations			
Bond lengths (Å)	0.003	0.003	0.004
Bond angles (°)	0.558	0.548	0.561
Validation			
MolProbity score	1.52	1.17	1.37
Clashscore	5.62	3.77	6.00
Poor rotamers (%)	0	0.52	1.13
Ramachandran plot			
Favoured (%)	96.64	98.30	98.29
Allowed (%)	3.36	1.70	1.71
Disallowed (%)	0	0	0

### Image processing

Single-particle analysis of the cryo-EM data was performed using cryoSPARC^30^. Particles were picked using a reference-free blob picker (minimum particle diameter, 100 Å; maximum particle diameter, 200 Å) followed by particle extraction (particle box size 440 pixels with 2x binning resulting in 220 pixels) and 2D classification. Initial promising 2D classes were used for template picking, followed by extraction and multiple rounds of 2D classification. For the wild-type protein, ab initio reconstruction was conducted, followed by heterogeneous refinement using well-resolved and poorly-resolved classes. Several rounds of non-uniform refinement and heterogeneous refinement were conducted until no further improvement was observed. 382,328 particles were chosen for non-uniform refinement with C4 symmetry, which yielded a map at 3.54 Å for apo Kir7.1. 556,510 particles from the Kir7.1-PIP_2_ dataset were chosen for non-uniform refinement with C4 symmetry, producing a map at 3.29 Å. For Kir7.1-R162W a 3D volume from the apo dataset was low-pass filtered to 10 Å and used as an initial volume in heterogenous refinement. A final 140,275 particles were selected for non-uniform refinement with C4 symmetry, yielding a map at 3.52 Å (Supplementary Figs. 3, 7 and 8).

### Model building and refinement

The protein models were initially built automatically using ModelAngelo^31^, and additional protein residues were added using COOT^32^. The resulting protein model was iteratively refined using ISOLDE^33^ and PHENIX^34^ and manually adjusted in COOT. For the structure of Kir7.1 in complex with PIP_2_, molecules of PIP_2_ were placed in the unmodelled density, and the acyl chains were manually deleted until only the portion of PIP_2_ within the density remained. PHENIX eLBOW was used to generate ligand restraints and iterative refinement cycles using PHENIX, ISOLDE and COOT were undertaken. For Kir7.1-R162W, the WT model was placed in the map, and residue 162 was mutated to tryptophan. The model was then refined as described above. The refinement statistics are shown in Table 1 and representative densities are shown in Supplementary Fig. 9.

### Structural analyses

Structural alignment and figures were made in UCSF ChimeraX^35^. Pore diameters between van der Waals surfaces for all models were calculated with HOLE2^36^ and visualised with visual molecular dynamics software^37^.

### NanoDSF

NanoDSF thermal unfolding curves were measured with a Prometheus Panta (NanoTemper) for purified Kir7.1 and Kir7.1-R162W. Measurements were conducted using the final size-exclusion chromatography fractions diluted to either 0.2 or 0.4 mg/ml in a buffer containing 0.02% GDN, 20 mM Tris-HCl, pH 7.5, 150 mM KCl. For Tm experiments testing Kir7.1-WT and Kir7.1-R162W, dioctanoyl (C8) lipid analogues were added to a final concentration of 25 μM. The lipids tested included phosphatidylserine (PS), phosphatidylcholine (PC), PI(4,5)P_2_, PI(3,4,5)P_3_ and PI(3,4)P_2_. Each experiment was performed in triplicate (three technical replicates). To assess the dose response for Kir7.1-WT to PI(4,5)P_2_, measurements were conducted under apo conditions (*n* = 4) and with the addition of C8-PI(4,5)P_2_ [0.1 (*n* = 5), 1 (*n* = 6), 10 (*n* = 3), 100 (*n* = 5)] μM. For Kir7.1-R162W compared to Kir7.1-WT the experiment was performed with three technical replicates and two repeats. Thermal measurements were carried out over a range from 20 to 90 °C with 1 °C min^−1^ steps. The data analysis was performed in the software PR. ThermControl (NanoTemper). ΔTm values were calculated as the difference in melting temperature between lipid-treated sample and apo Kir7.1.

### Statistics and reproducibility

Exact *n* values and definitions of replicates are provided in the corresponding figure legends. Measurement of Kir7.1-WT Tm in response to PIP_2_ was performed in a single experiment with four technical replicates (0 µM, 0.1 µM and 10 µM PIP_2_) or five technical replicates (1 µM and 100 µM PIP_2_). All other measurements of Kir7.1-WT and Kir7.1-R162W Tm in response to lipids were performed in two independent experiments, each with three technical replicates per condition.

Data were analysed using GraphPad Prism. Results are presented as mean ± standard deviation (SD), with individual data points shown and error bars representing SD.

### Reporting summary

Further information on research design is available in the Nature Portfolio Reporting Summary linked to this article.

## Supplementary information

Transparent Peer Review file

Supplementary Information

Description of Additional Supplementary Files

Supplementary Data 1

Reporting Summary

## Data Availability

The EM maps for Kir7.1-WT, Kir7.1-PIP_2_ and Kir7.1-R162W have been deposited in the EMDB under accession codes EMD-55878, EMD-55892 and EMD-55889, respectively. Corresponding atomic coordinates are deposited in the PDB with IDs 9TFV, 9TG6 and 9TG3, respectively. Uncropped and unedited gel images were provided as Supplementary Fig.10. Raw data for NanoDSF experiments were provided as Supplementary Data 1.
