# Supplementary material for: Cryo-EM structure of the human Kir7.1 channel reveals the molecular basis of snowflake vitreoretinal degeneration disease
Source: Commun Biol. 2026 Jun 21;9:1311. doi: 10.1038/s42003-026-10436-0 (PMC13646276; doi:10.1038/s42003-026-10436-0)
Supplement: Supplementary file 1 [file 42003_2026_10436_MOESM1_ESM.pdf]

nature portfolio

Peer Review File

Cryo-EM structure of the human Kir7.1 channel reveals the molecular basis of Snowflake Vitreoretinal Degeneration disease

Corresponding Author: Professor Bonnie Wallace

This file contains all reviewer reports in order by version, followed by all author rebuttals in order by version.

Version 0:

Reviewer comments:

Reviewer #1

(Remarks to the Author)
In this very interesting and well written paper, O'Malley et al. Present three structures of the Kir7.1 potassium channel: a) the apo Kir7.1, wild type, b) Kir7.1 in complex with PIP2 and c) the mutant Kir7.1-R162W. This mutant is responsible for the rare disease snowflake Vitreoretinal Degeneration Disease through a loss of channel function. Inwardly rectifying K^+ (Kir) channels are essential regulators of membrane potential and cellular excitability across diverse tissues.
NanoDSF measurements established that the binding of PIPs has a stabilizing effect on this potassium channel.

Comments:

1) Structure of the Kir7.1 in complex with PIP2: The binding of PIP2 widens the helix bundle crossing in Kir7.1. The G-loop gate, another region commonly constricted in Kir channels, remained dilated and open in both the apo and PIP2-bound states of Kir7.1. An interesting observation is that in contrast to some previously reported Kir channel structures, PIP_2-bound Kir7.1 did not exhibit substantial conformational changes in the cytoplasmic domain (CTD). The channel remained in an 'extended' conformation in both apo and PIP_2-bound states. In other Kir structures in complex with PIP2, the channel is in the compact form.
a) The detergent used for the extraction, purification and cryo-EM studies of the Kir7.1 is the GDN glycol-diosgenin. This is a steroid-derived detergent. It cannot be rule out that GDN may interact with the channel and prevent the compressed form. It has been reported that GDN binding could affect the conformation of protein (see Di Trani et al. doi: 10.1073/pnas.2205228119 "Structural basis of mammalian Comple IV inhibiton by steroids"). As mentioned by the authors, GDN was used for the study of Kir6.2 however the activation of this channel is completely different (through the SUR protein and not PIP2)
b) Another explanation would be that another activator is involved in the activation of the channel and the combined effect helps for the compact form.
c) The Kir channels are known for their high dynamics in solution. In Cryo-EM studies, the presence of various populations of different conformational states on the same grid was highlighted (for instance, Kir2.1). During the image analysis, many 3D classes are eliminated. Exploring them could provide information on the different conformations present in the same sample. Could a more compressed form be present in one of these eliminated classes? It would be interesting to investigate these discarded classes even at low resolution

2) Of the three structures shown, only one (extended state, apo Kir7.1) shows the presence of two potassium ions in the selectivity filter. Surprisingly the other structures do not show any potassium or any other ions inside the conductive pathway. Any explanation for this?

3) The study on the mutant is convincing and give a precise molecular mechanism by which R162W impairs Kir7.1 activity and contributes to retinal degeneration. The structures reveal that the R162W mutations disrupts ion conduction by positioning bulky tryptophan side chains within the channel pore, effectively blocking ion flow.

Reviewer #2

(Remarks to the Author)
This manuscript by O'Malley et al. reports the cryo-EM structures of the human Kir7.1 channel in the apo and PIP_2-bound states, as well as the retinal disease–associated R162W mutant in the apo state.
The authors show that the tryptophan side chain introduced by the R162W mutation protrudes into the channel pore, physically blocking K^+ permeation, while having little effect on binding of the essential ligand PIP_2.
Furthermore, they demonstrate that PIP_2 binding expands the helix bundle crossing (HBC) without triggering cytoplasmic domain (CTD) docking, suggesting an intermediate, Kir7.1-specific conformation.

The structural findings are novel and relevant to understanding Kir7.1 gating and the pathogenesis of retinal disease.
The cryo-EM data, although of moderate resolution, are of sufficient quality to support the authors' main structural claims.
However, the absence of electrophysiological recordings limits the direct correlation between structure and function, and therefore the proposed mechanisms remain speculative.
In addition, there are several critical inconsistencies between the text and figures (detailed below).

I therefore recommend major revisions before this work can be considered for publication in Communications Biology.

Major Comments
1.
No experimental data are shown in Fig. 2d, despite the description in lines 131–133.
2.
In lines 133–135, the authors should perform electrophysiological experiments on WT and mutant channels to confirm whether PIP2 binds at the position observed in the structure.

3.
In lines 188–191, the authors interpret the extended PIP_2-bound state as an intermediate or pre-activated state.
However, a previous biophysical study (PMID: 12525701) demonstrated that C8-PIP_2 (from Echelon), the same ligand used for structure determination here, can fully activate Kir7.1.
Without experimental evidence, such as correlating the degree of HBC expansion with conductance via electrophysiology, it is difficult to agree with the authors' interpretation that this structure represents an intermediate or pre-activated state.
If it is difficult to confirm this interpretation experimentally, the authors should at least tone down this claim in the manuscript.

4.
In lines 228–233, the authors report that the R162W mutation blocks the pore but has little effect on PIP2 binding based on thermal stability assays.
Electrophysiological experiments should be conducted to confirm that PIP_2 binding affinity is not substantially altered, for example using voltage-sensing phosphatase (VSP).
Although R162W exhibits a dominant-negative effect, approaches such as co-expression (as in PMID: 23255580) or generating a WT–R162W concatemer could allow evaluation of PIP2 binding affinity in the mutant.

5.
In Supplementary Figures 2 and 5, the panels and legends appear to be swapped.

6.
In Supplementary Table 1, despite having higher map resolution than the other structures, the PIP2-bound Kir7.1 structure contains almost half the number of non-hydrogen atoms.
The authors should verify whether this value is correct.

7.
Statistical tests should be applied to all applicable panels.

Minor comments
1.
In Fig. 3c, indicate the species origin of amino acids (e.g., T153).

2.
Include a figure illustrating the Kir conformational changes described in lines 169–180, for readers less familiar with the field.
Panels showing superimposition of both closed and PIP_2-bound Kir structures would be helpful.

Reviewer #3

(Remarks to the Author)
General comments:
In this manuscript, Niamh and colleagues present the cryo-EM structure of the human potassium channel 7.1 (Kir7.1) in its apo form, as well as an intermediate state with PIP2 bound, which is distinct from other Kir channels. Additionally, they determined the cryo-EM structure of a Kir7.1 variant bearing the Snowflake Vitreoretinal Degeneration (SVD) disease-associated R162W mutation, which provides valuable insights into the molecular mechanism of Kir7.1-R162W-induced SVD.

However, this study has significant limitations that reduce its impact and novelty, particularly in light of a similar preprint published on bioRxiv a year ago (Peisley et al., 2024, doi:10.1101/2024.06.07.597981).
A major strength of this current work is that the structural analysis of the R162W mutant provides an alternative mechanism for its pathogenicity. While previous studies hypothesized that the loss of the positive charge residue R162 disrupts PIP_2 binding, the current structure suggests that R162W can still bind with PIP2 at a compromised level, and the steric obstruction of the pore by tryptophan side chains is the primary defect. This represents a meaningful advance in understanding the molecular basis of Snowflake Vitreoretinal Degeneration.
Additionally, this paper expressed and purified the Apo-Kir7.1 and Kir7.1-PIP2 in the mammalian expression system (Expi293F), which may better reflect physiological conditions and post-translational modifications. Interestingly, the PIP_2-bound structure obtained here exhibits an open conformation, contrasting with the docked conformation observed in the insect cell-derived structures from the bioRxiv study. This difference may point to system-dependent conformational changes or an intermediate state relevant to channel gating.
The major weakness of this study is the absence of functional assays to support their key claims about the effect of PIP_2 on channel gating and mutation-induced blockage. Several figures are also incomplete or unclear, further limiting the interpretability of the results. Additionally, the author should further improve the quality of the figures. For example, Fig 4C does not show clear density for R162W, which is important for evaluating the map quality.

Specific Comments:
The overall structure of Kir7.1.
1. Figure 1 orientation labels are confusing and should be clarified.
2. In Figure S1a, the X-axis is overlaid with the volume label.
3. The local density for the glycosylation site (Asn95) should be clearly shown in the supplement (Fig. S3) to support the claim of glycosylation.
4. The results of 3D variability analysis (3DVA) are mentioned but not shown. These should be included to support claims about flexibility.
Kir7.1 in complex with PIP2.
1. Residue D43, which is stated to interact with PIP_2, is not labeled in Figure 2b.
2. “No direct interaction was observed between PIP2 and residues within the flexible TMD-CTD linker or the cytoplasmic domain.”. But in Figure 2d, some residues from the linker and CTD are highlighted in blue.
PIP2 binding widens the helix bundle crossing in Kir7.1.
1. Figures 3c and 3d have no corresponding context, and the helix tilt angles are not consistent, and lack clear metrics (e.g., distance measurements).
2. Beyond NanoDSF, no functional data (e.g., electrophysiology) are provided to demonstrate PIP_2-dependent activation of Kir7.1.
Comparison of Kir7.1-PIP2 with other activated Kir channels
1. A direct structural comparison with the cytoplasmic domains of other Kir channels is missing.
2. The extended discussion on conformational states (docked or extended) belongs in the Discussion, not the Results.
3. Figure S3 should highlight the Asn95 residue in the model, and better to show the local map density around Asn95.
R162W Mutant Analysis:
1. The tryptophan side chains should be clearly highlighted in Figure 4a.
2. “In the WT structure, this region is less well-resolved, and the arginine sidechains appear to extend outwards but are likely dynamic and flexible.” Where is the wild-type R162 sidechain density for comparison? And how can you be sure “R162W resides within the PI(4,5)P2 binding pocket, and it was of interest to investigate if this mutation would impact lipid binding.”?
3. Figure 4d should present the lipid concentration in the figure legend.
4. Figure S5 has the wrong figure legend sequence.
5. The NanoDSF data in Figure 4d do not directly measure PIP_2 binding affinity. A more direct assay (e.g., lipid binding or functional rescue) is needed to support the conclusion that PIP_2 binding is retained.

Discussion
1. The discussion of the R162W-induced SVD mechanism would benefit from a schematic diagram. Electrophysiological data are essential to confirm the proposed conduction block.
2. “Although PIP_2 binding may still occur in the mutant, the resultant conformational changes are insufficient to overcome the blockade created by the mutated residue.”. If the mutant retains PIP_2 binding, as claimed, a binding ability study or structure of R162W-PIP_2 should be attempted to assess conformational response.

Version 1:

Reviewer comments:

Reviewer #1

(Remarks to the Author)
After reading the first version of the manuscript, I made a few comments to which the authors responded in detail and convincingly.

I am satisfied with their responses. However, I would like a modification to the new Figure 5 concerning the visualization of K+ ions in the conduction pathway. I had already raised the question of the number and the location of the K+ in the conduction pathway in the three calculated proposed structures. Apo, compled wiyth PIP2 and the mutant . The answer of the authors was : "Based on the densities obtained by cryo-electron microscopy, we modeled two potassium ions in the apo-WT and R162W structures, and four ions in the structure bound to PIP_2." These ions should now be clearly and correctly visible in Figure 5, and if possible, in the other figures as well.

Reviewer #2

(Remarks to the Author)
I appreciate the authors' revisions, in particular the toned-down interpretation of the conformational changes induced by PIP2 binding.
I also appreciate the clarification that the electrophysiological properties of Kir7.1 and the R162W loss-of-function phenotype have been extensively reported in prior studies, and that direct electrophysiological assessment of PIP2 sensitivity is not straightforward. In this context, the authors' use of NanoDSF as a complementary biophysical approach is reasonable.

However, a key concern remains regarding the interpretation of the NanoDSF data and the resulting claim that the R162W mutant retains PIP2 binding (or interaction) in a manner comparable to WT.

First, in Fig. 2c the authors show that PIP2 stabilizes purified Kir7.1 in a dose-dependent manner. However, stabilization observed using NanoDSF alone does not demonstrate that the interaction occurs at the canonical PIP2-binding pocket observed in the PIP2-bound WT structure. It does not exclude the possibility of altered binding modes or non-specific lipid/micelle-driven stabilization.
Therefore, the authors should test whether the PIP2-induced ΔTm shift depends on the identified PIP2 pocket by using pocket-disrupting mutant(s) expected to weaken or abolish PIP2 binding. A marked reduction or loss of the PIP2-dependent stabilization in such mutant(s) would strongly support the use of NanoDSF as a complementary biophysical approach.

Furthermore, in Fig. 4d the authors present NanoDSF data at a single lipid concentration and conclude that R162W retains PIP2 binding similarly to WT. This does not allow comparison of apparent sensitivity between WT and R162W. Although I understand NanoDSF does not directly provide a true Kd, a PIP2 concentration–response for R162W (analogous to the WT dose-response in Fig. 2c) would enable a quantitative comparison of apparent sensitivity (e.g., an apparent EC50) and would greatly clarify to what extent R162W alters PIP2 interaction.

With these additions, the conclusions regarding the proposed pore-blocking mechanism of R162W would be much better supported, further strengthening the manuscript.

Therefore, I recommend further revision, as the additional control and the R162W concentration–response NanoDSF are important to substantiate the authors' claims.

Reviewer #3

(Remarks to the Author)
Summary:
The authors have revised their manuscript describing the cryo-EM structures of the human Kir7.1 channel in Apo, PIP2-bound, and R162W mutant forms. They propose a mechanism where the R162W mutation causes Snowflake Vitreoretinal Degeneration (SVD) through steric occlusion of the pore rather than loss of PIP2 affinity. Additionally, they propose that PIP2 binding opens the Helix Bundle Crossing (HBC) without docking the Cytoplasmic Domain (CTD), suggesting a novel activation intermediate distinct from the fully docked conformation reported in other Kir channels. They attribute this difference largely to the expression system and detergent conditions.
I have carefully reviewed the revised manuscript and the authors' responses to the previous comments. I appreciate the authors' efforts in improving the clarity of the figures and rewriting sections to address the concerns regarding the lack of functional data. I also acknowledge the inclusion of NanoDSF statistical analysis and the citation of historical electrophysiological data to contextualize the work.
However, while the analysis of the R162W mutant is structurally compelling and distinct from recent work in the field, the interpretation of the PIP2-bound wild-type structure remains a point of concern. The manuscript requires careful textual revision to avoid over-interpreting what is likely an experimentally derived conformational state as a definitive physiological "new active form".
Comments:
1. Interpretation of the PIP2-bound "Intermediate" State. In the revised manuscript and rebuttal, the authors argue that their structure (Mammalian cells/GDN) represents a more "native-like" or "intermediate" state compared to the Peisley et al. structure (Insect cells/DDM+CHS), which shows the canonical docked conformation. While I appreciate the authors' hypothesis, it is scientifically risky to claim the "extended" state is more physiological solely based on the expression system. In the broader context of Kir channel structural biology (e.g., Kir2.2, Kir3.2, Kir6.2), the docked conformation is widely accepted as the fully active state essential for channel gating.
It is equally plausible that the "extended" PIP2-bound state observed here represents a decoupled or desensitized state, possibly due to the loss of specific lipids during GDN purification that are required for the full CTD docking, rather than a functional "pre-activated" intermediate. The authors should balance their discussion further. Instead of implying the Peisley

structure is less native due to CHS/Insect cells, the authors should explicitly acknowledge that their structure might capture a state where the CTD-TMD coupling is weak or uncoupled, and that the "docked" state observed by Peisley et al. represents the fully activated endpoint. The current text (Discussion) leans too heavily on the "intermediate" narrative without sufficient functional proof.
2. The Mechanism of R162W vs. R162Q. I commend the authors for retaining the focus on the R162W mutation. This is the strongest point of differentiation from the R162Q structure. The mechanism of a steric block by the bulky Tryptophan side chain in the pore (R162W) is structurally distinct from the mechanism proposed for R162Q (which describe as stabilizing a docked but non-conductive state or altering gating kinetics). The R162Q mutation (Retinitis Pigmentosa) causes a gain-of-function, whereas R162W (SVD) causes a loss-of-function.
The authors should explicitly contrast their R162W structural mechanism with the R162Q mechanism described in the preprint in the Discussion section. Highlighting that different mutations at the same site can lead to disease via distinct structural mechanisms (steric block vs. uncoupling/locking) would significantly add value to this publication and justify its coexistence with the other paper.
3. Functional Validation Claims. In response to previous reviews, the authors declined to perform new electrophysiology, citing existing literature. While I accept that the basic function of Kir7.1 is established, the lack of functional data specific to the constructs and lipids used here creates a gap in correlating the structure to function. I appreciate that the authors have toned down the language regarding PIP2 "affinity" based on the NanoDSF data. The current conclusion that R162W does not abolish PIP2 interaction is reasonable. The authors should provide the text strictly adheres to "thermal stability" rather than "binding affinity".
4. Visualizing the Gating Cycle. The new schematic (Figure 5) helps summarize the authors' model. However, based on reviewer #1, the "Activated/Pre-activated" middle panel is potentially misleading if presented as the final conducting state. I suggest adding a visual note or question mark indicating that full activation (high conductance) likely requires the transition to the Docked state (as seen in other Kirs), to be consistent with the field's general consensus. This would make the model more robust and less susceptible to criticism that it ignores the canonical gating mechanism.

Version 2:

Reviewer comments:

Reviewer #2

(Remarks to the Author)
I appreciate the authors’ revisions and have no further suggestions.

Reviewer #3

(Remarks to the Author)
All my concerns have been addressed.

Open Access This Peer Review File is licensed under a Creative Commons Attribution 4.0 International License, which permits use, sharing, adaptation, distribution and reproduction in any medium or format, as long as you give appropriate credit to the original author(s) and the source, provide a link to the Creative Commons license, and indicate if changes were

made.
In cases where reviewers are anonymous, credit should be given to 'Anonymous Referee' and the source.
The images or other third party material in this Peer Review File are included in the article’s Creative Commons license, unless indicated otherwise in a credit line to the material. If material is not included in the article’s Creative Commons license and your intended use is not permitted by statutory regulation or exceeds the permitted use, you will need to obtain permission directly from the copyright holder.
To view a copy of this license, visit https://creativecommons.org/licenses/by/4.0/

Dear Editor and Reviewers,

We sincerely appreciate the constructive feedback and thoughtful comments provided by the reviewers. We have carefully revised the manuscript to improve its clarity and presentation, incorporating additional figures and re-written sections to address the major concerns raised.
As noted in our detailed responses, we have not repeated electrophysiological experiments because these have been comprehensively reported in previous studies. The key literature is summarised in our response to Reviewer 2. Our work builds upon this established functional understanding and provides a novel structural perspective that complements existing knowledge of Kir7.1 channel regulation.

Our point-by-point responses to the reviewers comments are provided below, the critiques are in *italics* and our responses are in blue, and the corresponding edits in the revised manuscript are highlighted in red.

Reviewer #1 (Remarks to the Author):

In this very interesting and well written paper, O'Malley et al. Present three structures of the Kir7.1 potassium channel: a) the apo Kir7.1, wild type, b) Kir7.1 in complex with PIP2 and c) the mutant Kir7.1-R162W. This mutant is responsible for the rare disease snowflake Vitreoretinal Degeneration Disease through a loss of channel function. Inwardly rectifying K^+ (Kir) channels are essential regulators of membrane potential and cellular excitability across diverse tissues.
NanoDSF measurements established that the binding of PIPs has a stabilizing effect on this potassium channel.

We thank the reviewer for their time and effort in evaluating our manuscript and for concisely summarising our work.

Comments:

1) Structure of the Kir7.1 in complex with PIP2: The binding of PIP2 widens the helix bundle crossing in Kir7.1. The G-loop gate, another region commonly constricted in Kir channels, remained dilated and open in both the apo and PIP2-bound states of Kir7.1. An interesting observation is that in contrast to some previously reported Kir channel structures, PIP_2 -bound Kir7.1 did not exhibit substantial conformational changes in the cytoplasmic domain (CTD). The channel remained in an 'extended' conformation in both apo and PIP_2 -bound states. In other Kir structures in complex with PIP2, the channel is in the compact form.
a) The detergent used for the extraction, purification and cryo-EM studies of the Kir7.1 is the GDN glycol-diosgenin. This is a steroid-derived detergent. It cannot be rule out that GDN may interact with the channel and prevent the compressed form. It has been reported that GDN binding could affect the conformation of protein (see Di Trani et al. doi: 10.1073/pnas.2205228119 "Structural basis of mammalian Comple IV inhibiton by steroids"). As mentioned by the authors, GDN was used for the study of Kir6.2 however the activation of this channel is completely different (through the SUR protein and not PIP2)

We thank the reviewer for raising this point. We agree that the potential influence of cholesterol or cholesterol-like molecules on Kir7.1 conformation is an important consideration. However, previous studies have shown that Kir7.1 activity is not downregulated by cholesterol (Rosenhouse-Dantsker et al., 2010, Channels 4(1):63–66), suggesting that cholesterol itself does not exert an inhibitory effect on this channel.

In addition, GIRK channel structures solved in DDM/CHS micelles have been observed in both docked and extended conformations (Niu et al., 2020, eLife 9:e60552). Similarly, the Kir7.1 structures reported in a recent preprint (doi: 10.1101/2024.06.07.597981) where the channel is captured in the docked conformation — were determined in LMNG/CHS. Since CHS is a well-established cholesterol analog that closely resembles the sterol structure, these findings collectively suggest that cholesterol or its mimetics do not inherently prevent CTD docking.

We fully agree, however, that the detergent environment can influence conformational states. We note that Kir6.2, which requires SUR subunits for activation but also depends on PIP_2 binding, adopts a docked conformation when purified in GDN micelles. This observation supports the view that GDN micelles do not intrinsically obstruct CTD docking, further supporting that the observed conformations may reflect genuine channel states rather than detergent artifacts.

In response to this comment, we have removed the direct comparison to Kir6.2 from the manuscript, as the activation mechanisms differ, and have added a statement acknowledging that experimental conditions can influence the observed conformations, an important consideration for all Kir channel structures discussed in this study.

b) Another explanation would be that another activator is involved in the activation of the channel and the combined effect helps for the compact form.
We agree with the reviewer, and we have always considered this a possibility. We have now included mention of this in the text (lines 301-302).

c) The Kir channels are known for their high dynamics in solution. In Cryo-EM studies, the presence of various populations of different conformational states on the same grid was highlighted (for instance, Kir2.1). During the image analysis, many 3D classes are eliminated. Exploring them could provide information on the different conformations present in the same sample. Could a more compressed form be present in one of these eliminated classes? It would be interesting to investigate these discarded classes even at low resolution.
We appreciate the reviewers comment, and this was something we were very aware of during data processing. A substantial amount of time was spent specifically in search of any docked class(es). Some approaches involved several applications of 3DVA in cryoSPARC, 3D classification and heterogenous refinements. At no stage did we observe any volumes that could suggest a docked class. Since the conformational change upon docking is quite significant, we believe that it would've been detectable using at least one of these approaches. At one point to ensure we were not missing any classes we imported the GIRK2 cryo-EM map (EMDB:22200)

and used its projections for template-based picking. Although it can introduce some bias, even this targeted search did not detect any docked classes, indicating that these conformations were not present in the dataset.

2) Of the three structures shown, only one (extended state, apo Kir7.1) shows the presence of two potassium ions in the selectivity filter. Surprisingly the other structures do not show any potassium or any other ions inside the conductive pathway. Any explanation for this?
We thank the reviewer for noting this point. The apparent absence of potassium ions in two of the structures is solely due to the visualisation choices used in the figures. The apo WT structure was displayed in ribbon format, while the PIP_2 -bound and R162W structures were shown with the HOLE profile through the pore. For this analysis, the potassium ions had to be temporarily removed to allow the HOLE calculation, as their presence blocks the pathfinding algorithm. Based on the cryo-EM densities, we have in fact modelled two potassium ions in both the apo-WT and R162W structures, and four ions in the PIP_2 -bound structure.

3) The study on the mutant is convincing and give a precise molecular mechanism by which R162W impairs Kir7.1 activity and contributes to retinal degeneration. The structures reveal that the R162W mutations disrupts ion conduction by positioning bulky tryptophan side chains within the channel pore, effectively blocking ion flow.
We thank the reviewer for their comments and time to review our manuscript.

Reviewer #2 (Remarks to the Author):

This manuscript by O'Malley et al. reports the cryo-EM structures of the human Kir7.1 channel in the apo and PIP_2 -bound states, as well as the retinal disease–associated R162W mutant in the apo state.
The authors show that the tryptophan side chain introduced by the R162W mutation protrudes into the channel pore, physically blocking K^+ permeation, while having little effect on binding of the essential ligand PIP_2 .
Furthermore, they demonstrate that PIP_2 binding expands the helix bundle crossing (HBC) without triggering cytoplasmic domain (CTD) docking, suggesting an intermediate, Kir7.1-specific conformation.

The structural findings are novel and relevant to understanding Kir7.1 gating and the pathogenesis of retinal disease.
The cryo-EM data, although of moderate resolution, are of sufficient quality to support the authors' main structural claims.
However, the absence of electrophysiological recordings limits the direct correlation between structure and function, and therefore the proposed mechanisms remain speculative.
In addition, there are several critical inconsistencies between the text and figures (detailed below).

I therefore recommend major revisions before this work can be considered for publication in Communications Biology.

We thank the reviewer for their time and effort in evaluating our manuscript and for their comments which we have hopefully addressed below.

Major Comments

1.

No experimental data are shown in Fig. 2d, despite the description in lines 131–133.

We apologise and thank the reviewer for catching this error. This has now been fixed.

2.

In lines 133–135, the authors should perform electrophysiological experiments on WT and mutant channels to confirm whether PIP2 binds at the position observed in the structure.

We thank the reviewer for this valuable suggestion. We agree that linking PIP₂ binding to channel function is a key question for Kir7.1 biology. However, the electrophysiological properties of Kir7.1 and the functional consequences of the R162W mutation have already been extensively characterised in several independent studies. These works provide a comprehensive functional framework that our structural findings now directly complement.

Rohács et al. (2003) first demonstrated Kir7.1 activation by C8-PIP₂ using patch-clamp electrophysiology (Fig. 2; DOI: 10.1073/pnas.0236364100), and Pattnaik and Hughes (2009) further showed that Kir7.1 channels in the retinal pigment epithelium are activated and regulated by PIP₂ and C8-PIP₂ (DOI: 10.1152/ajpcell.00250.2009).

Multiple groups have since confirmed that the Kir7.1-R162W mutant is non-functional: Pattnaik et al. (2013) observed a complete loss of current in HEK cells (Figs. 2–3; DOI: 10.1371/journal.pone.0071744), and Zhang et al. (2013) reported the same phenotype in MDCK cells (Fig. 1; DOI: 10.1152/ajpcell.00363.2012). Vera et al. (2021) systematically investigated residue 162 and showed that increasing side-chain volume progressively diminishes channel activity (Fig. 4; DOI: 10.1113/JP280681). Importantly, the R162A substitution, removing the positive charge at this position, retained wild-type-like activity (Fig. 1D; DOI: 10.1113/JP280681), indicating that a positively charged side chain is not essential for PIP₂-mediated activation.

Collectively, these electrophysiological studies have already established both the requirement of PIP₂ for Kir7.1 activation and the non-conductive nature of the R162W mutant. Performing additional electrophysiology would therefore likely reproduce well-documented findings without providing additional mechanistic insight. Instead, our structural analysis reveals how PIP₂ engages Kir7.1 and rationalizes

the previously observed electrophysiological behaviours. Thus, our work complements and extends existing functional data.

3.
In lines 188–191, the authors interpret the extended PIP_2 -bound state as an intermediate or pre-activated state.
However, a previous biophysical study (PMID: 12525701) demonstrated that C8-PIP_2 (from Echelon), the same ligand used for structure determination here, can fully activate Kir7.1.
Without experimental evidence, such as correlating the degree of HBC expansion with conductance via electrophysiology, it is difficult to agree with the authors' interpretation that this structure represents an intermediate or pre-activated state.
If it is difficult to confirm this interpretation experimentally, the authors should at least tone down this claim in the manuscript.

We thank the reviewer for this thoughtful comment and agree that, without direct functional correlation, the structural data alone cannot definitively distinguish between an active or pre-activated state.

We note, however, that while electrophysiological measurements can assess overall channel conductance, they cannot directly report on the extent of HBC expansion or its correspondence to a specific structural conformation. The relationship between conformational states observed by cryo-EM and electrophysiological activity is therefore indirect.

Our interpretation of the extended PIP_2 -bound state as a potential pre-activated conformation was based on the relative displacement of the cytoplasmic domain compared to the transmembrane region, which may represent an intermediate state, potentially one that requires an additional binding partner for full CTD engagement (as noted by Reviewer 1). The structure presented in the preprint (doi: 10.1101/2024.06.07.597981) cannot be described as definitively active any more than the structure reported here, as the primary difference lies in the extent of cytoplasmic domain engagement. Importantly, it remains unclear whether this conformational change directly correlates with ion conductance. The pore diameter observed in our structure could, in principle, permit conduction. We have therefore revised the manuscript to present a more balanced interpretation that acknowledges both possibilities.

4.
In lines 228–233, the authors report that the R162W mutation blocks the pore but has little effect on PIP2 binding based on thermal stability assays.
Electrophysiological experiments should be conducted to confirm that PIP_2 binding affinity is not substantially altered, for example using voltage-sensing phosphatase (VSP).
Although R162W exhibits a dominant-negative effect, approaches such as co-expression (as in PMID: 23255580) or generating a WT–R162W concatemer could allow evaluation of PIP2 binding affinity in the mutant.
We thank the reviewer for this thoughtful suggestion regarding direct measurement of PIP_2 binding affinity. Unfortunately, electrophysiological approaches are not

suitable for assessing PIP_2 binding in the R162W mutant because this variant is completely non-conductive, as demonstrated by multiple prior studies (Pattnaik et al., 2013; Zhang et al., 2013; Vera et al., 2021). In the absence of measurable currents, methods such as VSP-based assays cannot report on PIP_2 sensitivity.
While co-expression with wild-type subunits or construction of WT–R162W concatemers could, in principle, restore measurable conductance, the resulting currents would arise primarily from functional WT subunits. As a result, any apparent PIP_2 responsiveness would reflect WT channel activity rather than the binding properties of the mutant, making such experiments difficult to interpret.
Given these constraints, we chose to assess PIP_2 interaction indirectly using NanoDSF thermal stability assays, which provide a direct biophysical measure of protein stabilisation upon lipid binding. Although we agree that this method does not fully substitute for electrophysiological validation, it represents the only experimentally tractable approach to probe PIP_2 association in the non-conductive mutant. To reflect this limitation, we have tempered our interpretation of the NanoDSF data in the revised manuscript.

5.
In Supplementary Figures 2 and 5, the panels and legends appear to be swapped.
We apologise for this mistake and thank the reviewer for noticing it. This has now been fixed.

6.
In Supplementary Table 1, despite having higher map resolution than the other structures, the PIP2-bound Kir7.1 structure contains almost half the number of non-hydrogen atoms.
The authors should verify whether this value is correct.
We thank the reviewer for spotting this and apologise for the error. This was due to hydrogen atoms being included for the other two models. This has now been fixed.

7.
Statistical tests should be applied to all applicable panels.
We have now included statistical tests for the NanoDSF data and significant results are shown on the figures. Statistical significance was not shown for all combinations for the PIP2 dose response as this made the figure difficult to interpret.

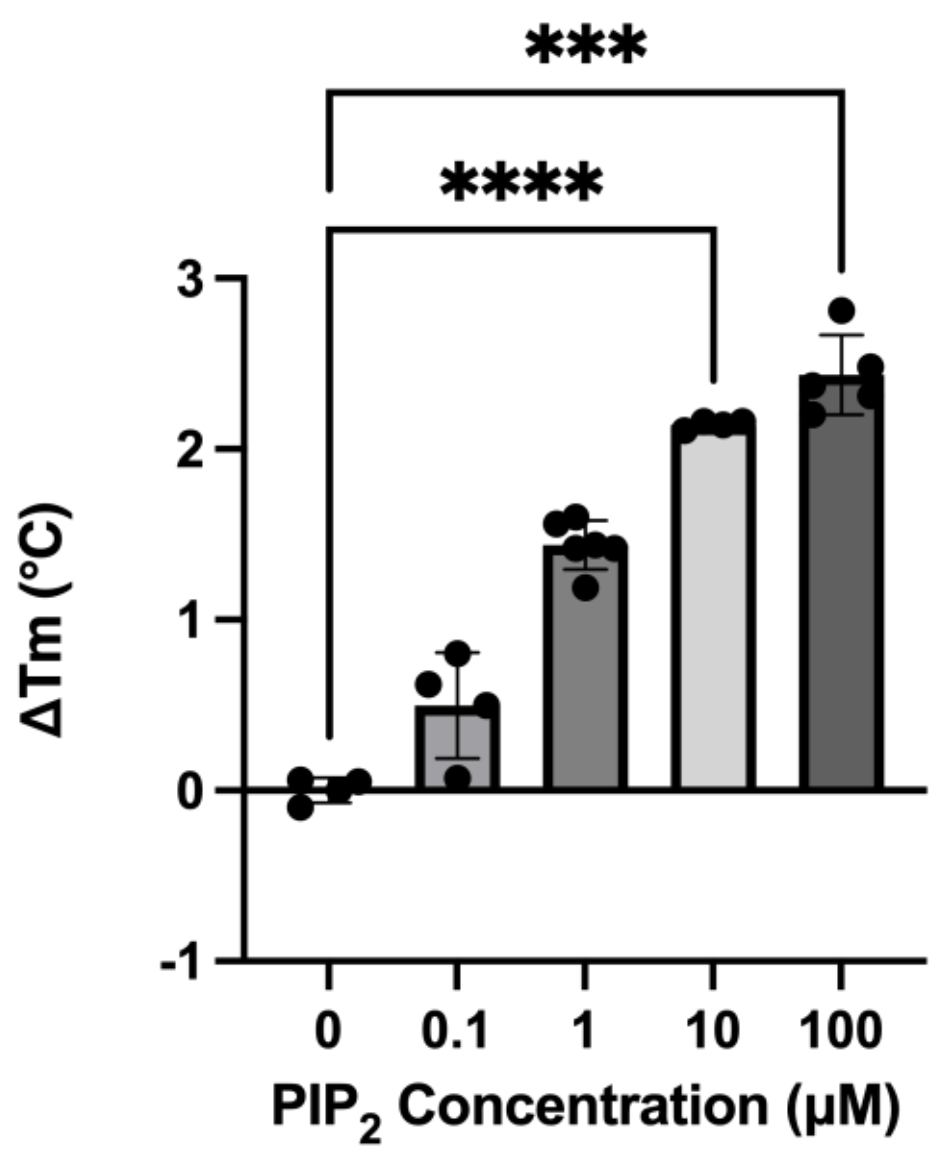

Fig. 2: c Thermal stability of Kir7.1 in response to increasing concentrations of PI(4,5)P_2 measured by NanoDSF. ΔTm values were calculated relative to apo-Kir7.1. Bars represent the mean, and error bars indicate standard deviation from at least three independent experiments. Statistical significance is shown only for comparisons between 0, 10, and 100 µM PI(4,5)P_2 for clarity (***$p < 0.001$; ****$p < 0.0001$).

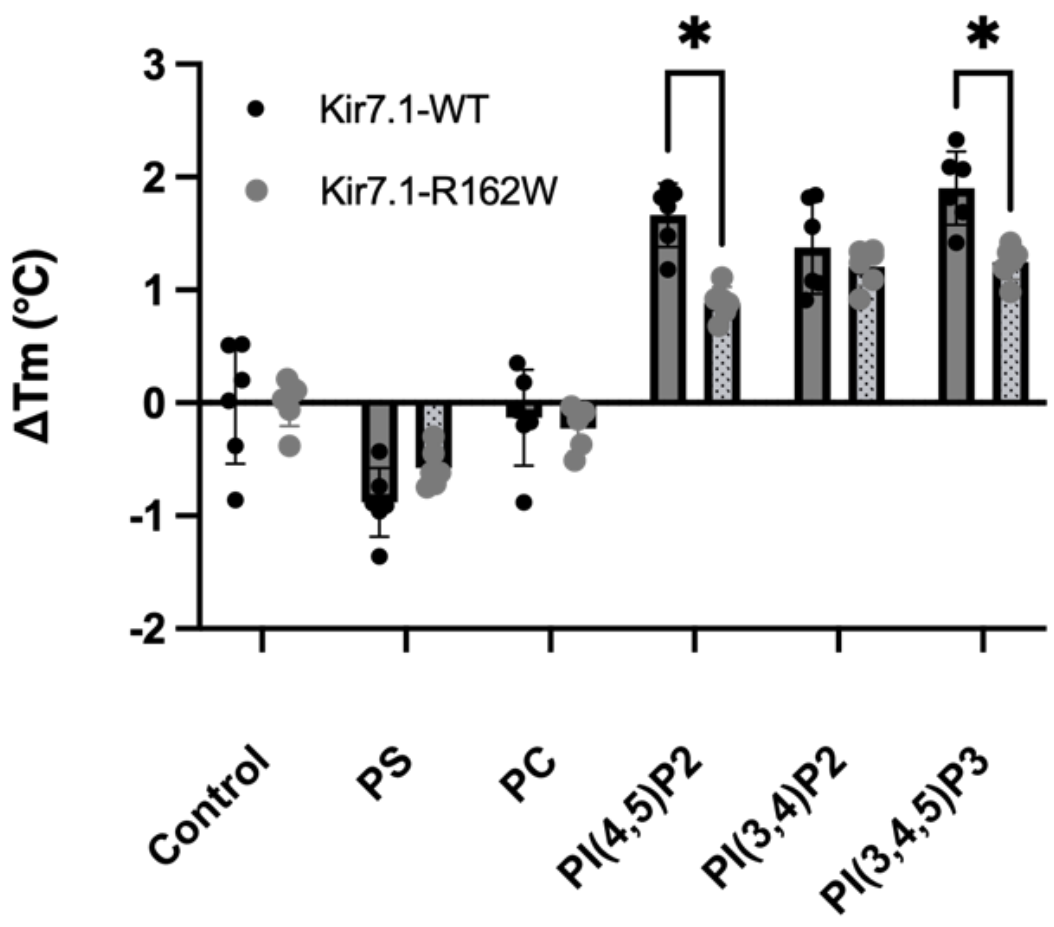

Fig. 4: d NanoDSF experiment measuring change in Tm of Kir7.1-WT and Kir7.1-R162W in the presence of various lipids. Lipids were added at a final concentration of 25 µM. Control measurements contained protein only. WT is shown in solid colour and R162W mutant in spots. An increase in Tm was observed for the PIPs but not for PS and PC. Two independent experiments were performed, each including three technical replicates per condition. Statistical significance was assessed using multiple unpaired t-tests, with significant differences indicated by asterisks.

Minor comments

1.

In Fig. 3c, indicate the species origin of amino acids (e.g., T153).

This has been included.

2.

Include a figure illustrating the Kir conformational changes described in lines 169–180, for readers less familiar with the field.

Panels showing superimposition of both closed and PIP_2 -bound Kir structures would be helpful.

We have included supplementary figures 6 and 7 to try to summarise the published structures. Supplementary figure 6 summarises Kir2.2 and Kir3.2 docked and extended structures, showing a similar widening of the HBC in both. Supplementary figure 7 outlines the variety of Kir3.2 structure – showing a docked crystal structure in the absence of PIP2.

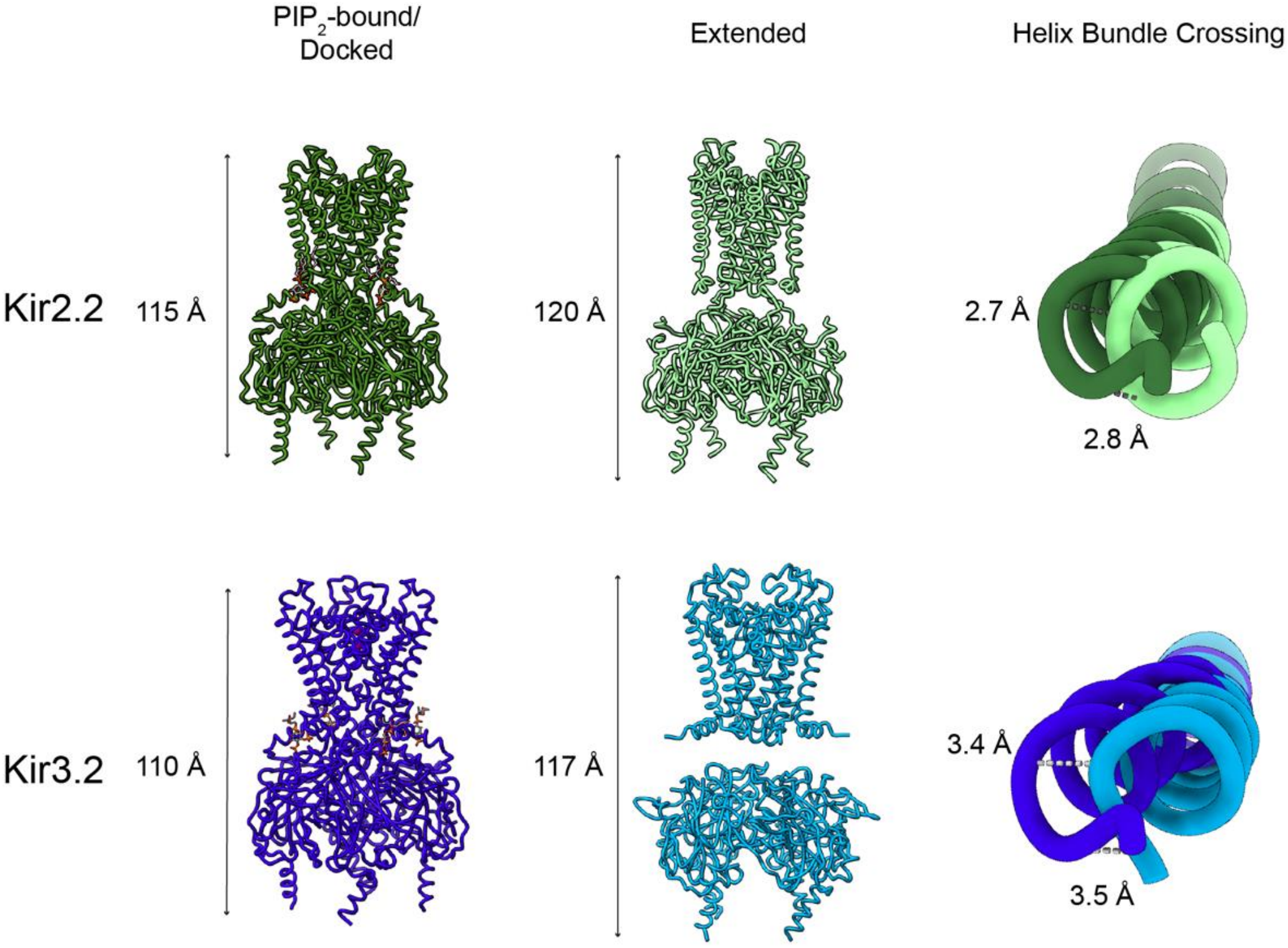

Supplementary Figure 6. Conformational differences between PIP_2-bound and extended states of Kir2.2 and Kir3.2 channels. Left: PIP_2-bound conformations of Kir2.2 and Kir3.2 show the cytoplasmic domain positioned closer to the membrane (Kir2.2: ~115 Å; Kir3.2: ~110 Å). **Middle:** In the extended conformations, the cytoplasmic domain disengages from the transmembrane domain, increasing the overall channel height (Kir2.2: ~120 Å; Kir3.2: ~117 Å). **Right:** Close-up view of the helix bundle crossing (HBC) from the intracellular side. In both channels, the inner transmembrane helix bends outwards around the conserved hinge glycine, resulting in a widened HBC and an expanded pore diameter. Distances were measured between the Cα of corresponding residues using ChimeraX. Models used: Kir2.2 (PDB: 3SPI, 3JYC) and Kir3.2 (PDB: 6XIT, 6XIS).

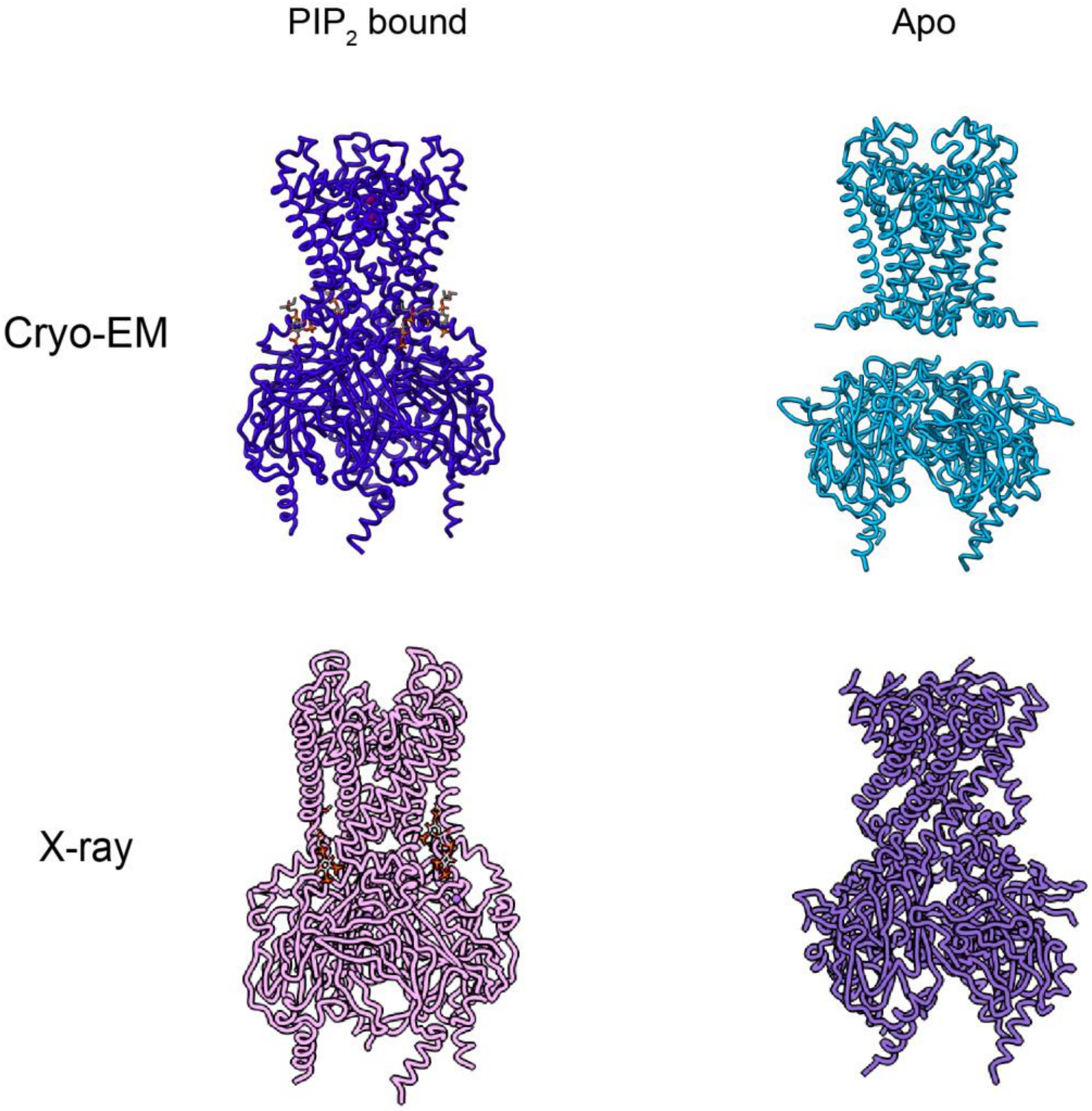

Supplementary Figure 7. Comparison of Kir3.2 Structures determined using Cryo-EM and X-ray crystallography in PIP_2-bound and apo states. Top: Cryo-EM structures of PIP_2-bound (6XIT) and Apo (6XIS) conformations of Kir3.2. **Bottom:** X-ray crystalographic structures of Kir3.2 in PIP_2-bound (3SYA) and Apo forms (3SYO).

Reviewer #3 (Remarks to the Author):

General comments:
In this manuscript, Niamh and colleagues present the cryo-EM structure of the human potassium channel 7.1 (Kir7.1) in its apo form, as well as an intermediate state with PIP2 bound, which is distinct from other Kir channels. Additionally, they determined the cryo-EM structure of a Kir7.1 variant bearing the Snowflake Vitreoretinal Degeneration (SVD) disease-associated R162W mutation, which provides valuable insights into the molecular mechanism of Kir7.1-R162W-induced SVD.
However, this study has significant limitations that reduce its impact and novelty, particularly in light of a similar preprint published on bioRxiv a year ago (Peisley et al., 2024, doi:10.1101/2024.06.07.597981).
A major strength of this current work is that the structural analysis of the R162W mutant provides an alternative mechanism for its pathogenicity. While previous studies hypothesized that the loss of the positive charge residue R162 disrupts PIP_2

binding, the current structure suggests that R162W can still bind with PIP2 at a compromised level, and the steric obstruction of the pore by tryptophan side chains is the primary defect. This represents a meaningful advance in understanding the molecular basis of Snowflake Vitreoretinal Degeneration.
Additionally, this paper expressed and purified the Apo-Kir7.1 and Kir7.1-PIP2 in the mammalian expression system (Expi293F), which may better reflect physiological conditions and post-translational modifications. Interestingly, the PIP_2 -bound structure obtained here exhibits an open conformation, contrasting with the docked conformation observed in the insect cell-derived structures from the bioRxiv study. This difference may point to system-dependent conformational changes or an intermediate state relevant to channel gating.
The major weakness of this study is the absence of functional assays to support their key claims about the effect of PIP_2 on channel gating and mutation-induced blockage. Several figures are also incomplete or unclear, further limiting the interpretability of the results. Additionally, the author should further improve the quality of the figures. For example, Fig 4C does not show clear density for R162W, which is important for evaluating the map quality.
We thank reviewer 3 for their thoughtful comments and for taking the time to effectively evaluate our manuscript.

Specific Comments:
The overall structure of Kir7.1.
1. Figure 1 orientation labels are confusing and should be clarified.
We agree with this point and these have now been changed and are clearer.

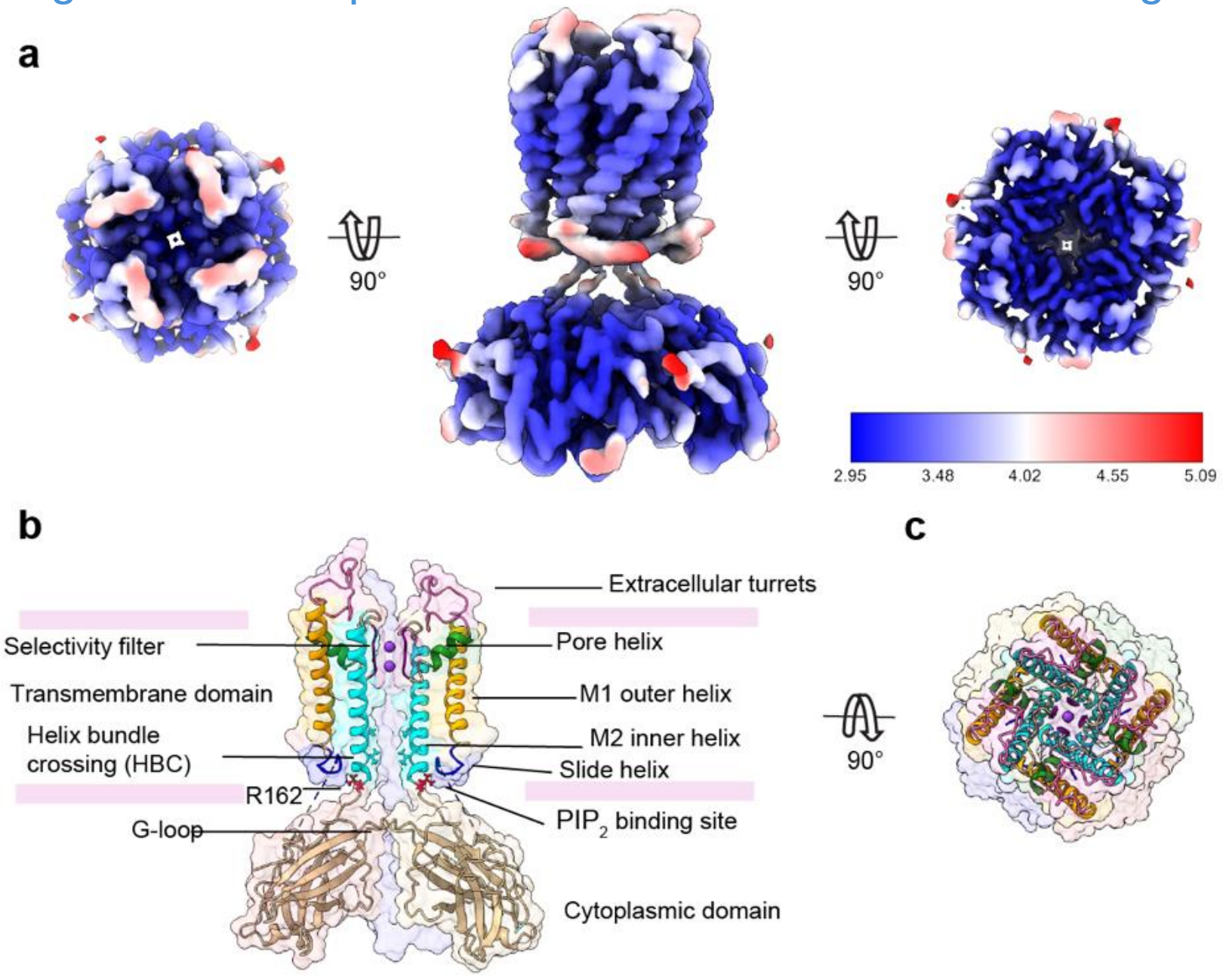

2. In Figure S1a, the X-axis is overlaid with the volume label.
This has been fixed.

3. The local density for the glycosylation site (Asn95) should be clearly shown in the supplement (Fig. S3) to support the claim of glycosylation.
This figure has been remade to improve clarity (see below).

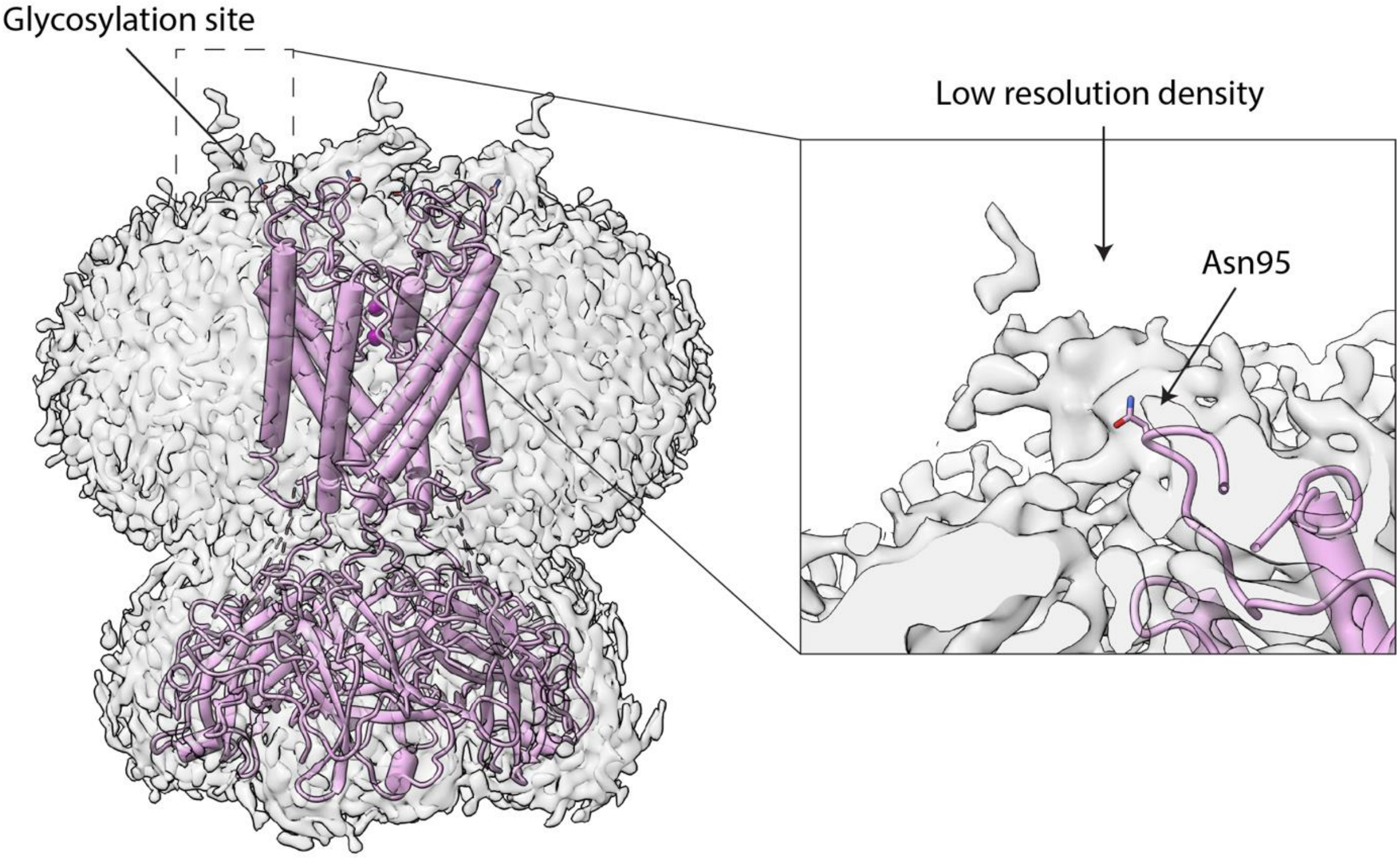

4. The results of 3D variability analysis (3DVA) are mentioned but not shown. These should be included to support claims about flexibility.
We have attached a powerpoint with the appropriate morph movies. These can be added as supplementary movies if thought to be helpful.

Kir7.1 in complex with PIP2.
1. Residue D43, which is stated to interact with PIP_2, is not labeled in Figure 2b.
This was an error and has been removed.

2. "No direct interaction was observed between PIP2 and residues within the flexible TMD-CTD linker or the cytoplasmic domain.". But in Figure 2d, some residues from the linker and CTD are highlighted in blue.
We apologise for this misleading wording The residues highlighted in blue have been shown to interact with PIP2 in other Kir channel published structures. The wording has been amended to clarify.

PIP2 binding widens the helix bundle crossing in Kir7.1.
1. Figures 3c and 3d have no corresponding context, and the tilt angles are not consistent, and lack clear metrics (e.g., distance measurements).
This is included now and the figure has been remade.

2. Beyond NanoDSF, no functional data (e.g., electrophysiology) are provided to demonstrate PIP_2 -dependent activation of Kir7.1.

There have been several published studies outlining Kir7.1 activation by PIP_2 (outlined above in response to reviewer 2). Given this extensive body of functional evidence, we did not repeat these electrophysiological experiments, as they would not provide additional mechanistic insight beyond what has already been firmly established in the literature. Instead, our study focuses on providing the structural basis for this known PIP_2 -dependent activation.

Comparison of Kir7.1-PIP2 with other activated Kir channels

1. A direct structural comparison with the cytoplasmic domains of other Kir channels is missing.

A summary of the published Kir2.2 and Kir3.2 structures is included in supplementary figures 6 and 7. These are included above in response to reviewer 2.

2. The extended discussion on conformational states (docked or extended) belongs in the Discussion, not the Results.

We agree with this comment and this has been moved.

3. Figure S3 should highlight the Asn95 residue in the model, and better to show the local map density around Asn95.

This figure has been remade to improve clarity (shown above).

R162W Mutant Analysis:

1. The tryptophan side chains should be clearly highlighted in Figure 4a.

This has now been included in Figure 4.

2. "In the WT structure, this region is less well-resolved, and the arginine sidechains appear to extend outwards but are likely dynamic and flexible." Where is the wild-type R162 sidechain density for comparison?

We thank the reviewer for this observation. In the wild-type structure, the resolution in the flexible linker region surrounding R162 is lower, and we therefore cannot confidently assign the exact position or orientation of the arginine side chain. As such, we did not feel it appropriate to draw a direct comparison between the WT and R162W models. We have revised the wording in the manuscript to avoid implying a defined side chain placement in the WT structure. Notably, the density corresponding to the tryptophan side chain in the R162W structure is clearly resolved, representing the most apparent difference between the two models.

And how can you be sure "R162W resides within the PI(4,5)P2 binding pocket, and it was of interest to investigate if this mutation would impact lipid binding."?

We appreciate the reviewer's question regarding our statement that "R162W resides within the PIP_2 binding pocket." This statement was based on previously published literature of the PIP_2 binding pocket being well characterised amongst Kir channels. The flexible TMD-CTD linker has been shown to form contacts with PIP_2 in Kir2.2 and Kir3.2. Specifically, residues homologous to R162 (R186 in Kir2.2) have been shown to make direct contacts to PIP_2.

Our experiment was designed in line with the published hypothesis that the disease-associated R162W mutation disrupts lipid binding. Importantly, this experimental

design preceded the determination of Kir7.1 wild-type structures at sufficient resolution to reveal the precise orientation or position of R162 and the bound PIP_2 molecules.

3. Figure 4d should present the lipid concentration in the figure legend.
This has now been included.

4. Figure S5 has the wrong figure legend sequence.
We thank the reviewer for spotting this error and this has now been fixed.

5. The NanoDSF data in Figure 4d do not directly measure PIP_2 binding affinity. A more direct assay (e.g., lipid binding or functional rescue) is needed to support the conclusion that PIP_2 binding is retained.
We appreciate the reviewer's comment and agree that NanoDSF does not directly measure PIP_2 binding affinity. We have therefore moderated our wording regarding the conclusion that PIP_2 binding is retained. Nonetheless, we consider the observed thermal stability profile an interesting finding that may suggest preserved lipid interaction, and we agree that additional, more direct assays would be valuable in future studies.

Discussion
1. The discussion of the R162W-induced SVD mechanism would benefit from a schematic diagram. Electrophysiological data are essential to confirm the proposed conduction block.
We have included figure 5 which is a schematic summary of the paper which should hopefully help the reader (included below). Electrophysiological data has been published on Kir7.1 showing complete loss of conductance and we don't believe repeating this would contribute to the field or confirm that the loss of function is due to a blockage. We have re-written the section to better highlight this published work.

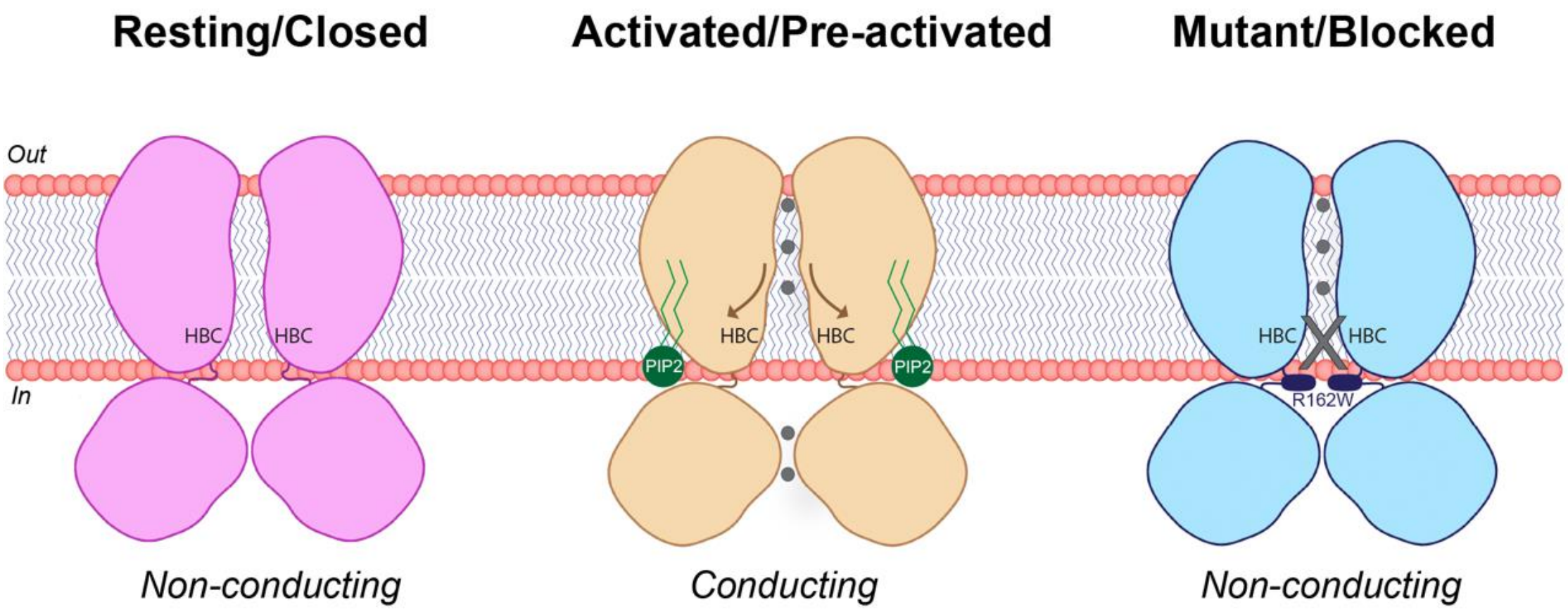

Figure 5. Schematic summary of Kir7.1 activation and pore blockage by R162W mutation. Left: Kir7.1 in the resting (closed) state in the absence of the activating lipid PIP_2. **Middle**: PIP_2-bound activated (or pre-activated) state with dilation at the helix bundle crossing. **Right**: The Snowflake Vitreoretinal Degeneration-associated

R162W mutation introduces a steric block within the channel pore, preventing ion conductance. HBC: Helix bundle crossing.

2. "Although PIP_2 binding may still occur in the mutant, the resultant conformational changes are insufficient to overcome the blockade created by the mutated residue.". If the mutant retains PIP_2 binding, as claimed, a binding ability study or structure of R162W-PIP_2 should be attempted to assess conformational response.

We appreciate the reviewer's thoughtful comment and agree that a structure of R162W bound to PIP_2 would be an ideal way to assess the conformational response. However, obtaining such a structure would require additional extensive experimental work beyond the current scope of this study. We have therefore moderated our language, as it is not possible to directly confirm PIP_2 binding by the mutant.
Previous studies have shown that the R162W channel is non-conductive even in the presence of PIP_2, indicating that, even if some binding were retained, it is insufficient to support channel activation. Based on our wild-type structures, the conformational effect of PIP_2 binding appears relatively modest and thus unlikely to compensate for the substantial pore constriction introduced by the R162W substitution.

We hope that our detailed responses and the accompanying revisions satisfactorily address all reviewer concerns. We believe the revised manuscript has been significantly improved and will be of interest to readers of *Communications Biology*.

Dear Editor and Reviewers,

We sincerely thank the editor and reviewers for the time and care taken to evaluate the revised version of our manuscript. We greatly appreciate the constructive feedback and have carefully revised the manuscript to address the points raised. Our detailed responses to each comment are provided below.

In addition, we have updated the order of the supplementary figures so that they now correspond to their citation order in the main text. We have also included a new Supplementary Figure 5 presenting the requested NanoDSF PIP2 dose–response experiment.

Reviewer comments are reproduced below, with our response are in blue and the corresponding edits in the manuscript are highlighted in red. We believe these revisions have strengthened the manuscript and addressed the reviewers' concerns.

Reviewer #1 (Remarks to the Author):

After reading the first version of the manuscript, I made a few comments to which the authors responded in detail and convincingly.

I am satisfied with their responses. However, I would like a modification to the new Figure 5 concerning the visualization of K+ ions in the conduction pathway. I had already raised the question of the number and the location of the K+ in the conduction pathway in the three calculated proposed structures. Apo, compled wiyth PIP2 and the mutant . The answer of the authors was : "Based on the densities obtained by cryo-electron microscopy, we modeled two potassium ions in the apo-WT and R162W structures, and four ions in the structure bound to PIP_2." These ions should now be clearly and correctly visible in Figure 5, and if possible, in the other figures as well.

We thank the reviewer very much for their comments. This is a helpful suggestion, and we have now updated Figure 5 to clearly display the potassium ions present in each structure. Specifically, the ions modelled in the apo-WT and R162W structures (two ions) and in the PIP2-bound structure (four ions), as described in the text, are now shown.

Reviewer #2 (Remarks to the Author):

I appreciate the authors' revisions, in particular the toned-down interpretation of the conformational changes induced by PIP2 binding.
I also appreciate the clarification that the electrophysiological properties of Kir7.1 and the R162W loss-of-function phenotype have been extensively reported in prior studies, and that direct electrophysiological assessment of PIP2 sensitivity is not straightforward. In this context, the authors' use of NanoDSF as a complementary biophysical approach is reasonable.

However, a key concern remains regarding the interpretation of the NanoDSF data

and the resulting claim that the R162W mutant retains PIP2 binding (or interaction) in a manner comparable to WT.

First, in Fig. 2c the authors show that PIP2 stabilizes purified Kir7.1 in a dose-dependent manner. However, stabilization observed using NanoDSF alone does not demonstrate that the interaction occurs at the canonical PIP2-binding pocket observed in the PIP2-bound WT structure. It does not exclude the possibility of altered binding modes or non-specific lipid/micelle-driven stabilization.
Therefore, the authors should test whether the PIP2-induced ΔTm shift depends on the identified PIP2 pocket by using pocket-disrupting mutant(s) expected to weaken or abolish PIP2 binding. A marked reduction or loss of the PIP2-dependent stabilization in such mutant(s) would strongly support the use of NanoDSF as a complementary biophysical approach.
We thank the reviewer for this important point. We agree that NanoDSF-based thermal stabilisation does not directly demonstrate that PIP2 binds at the known binding pocket, nor does it exclude the possibility of alternative lipid interactions or non-specific stabilisation effects. Whilst generating additional pocket-disrupting mutants would be a valuable approach to address this question directly, we were unfortunately unable to generate such constructs within the timeframe of this revision.

To address the reviewer's concern, we have revised the manuscript to clarify that that the NanoDSF experiments do not provide direct evidence that PIP2 binds to the same site in the mutant as in the WT structure. To highlight this we have included the line "This showed that the R162W mutation significantly reduced the stabilising effect of PI(4,5)P2 but did not abolish it. As NanoDSF reports on thermal stabilisation rather than direct lipid binding, these observations may reflect either weakened PIP2 interaction or reduced coupling between lipid engagement and conformational stabilisation of the channel. " The dose-response experiment (below) shows that the mutant protein exhibited a qualitatively similar response to PIP2 compared to WT, albeit with a reduced stabilisation. This is consistent with the possibility that PIP2 stabilises both proteins through a related mechanism, but we now explicitly acknowledge in the revised text that NanoDSF alone cannot definitively establish the binding mode.

Furthermore, in Fig. 4d the authors present NanoDSF data at a single lipid concentration and conclude that R162W retains PIP2 binding similarly to WT. This does not allow comparison of apparent sensitivity between WT and R162W. Although I understand NanoDSF does not directly provide a true Kd, a PIP2 concentration–response for R162W (analogous to the WT dose-response in Fig. 2c) would enable a quantitative comparison of apparent sensitivity (e.g., an apparent EC50) and would greatly clarify to what extent R162W alters PIP2 interaction.

We appreciate the reviewers comments and suggestions. In response, we have now conducted the NanoDSF experiment to directly compare the thermal stabilisation of WT and the R162W mutant in a dose response to PIP2. The results showed that the R162W mutant exhibited a similar dose-dependent increase in thermal stability, but with a markedly reduced ΔTm compared to WT.

We agree that NanoDSF does not provide a direct measure of lipid binding affinity and therefore cannot be used to determine a true binding constant. Accordingly, we have revised the manuscript to avoid interpreting these data in terms of binding affinity. The reduced stabilising effect observed for the mutant could reflect either weakened PIP2 interaction or reduced coupling between PIP2 engagement and conformational stabilisation of the channel. As NanoDSF cannot distinguish between these possibilities, we have clarified this limitation in the revised manuscript text.

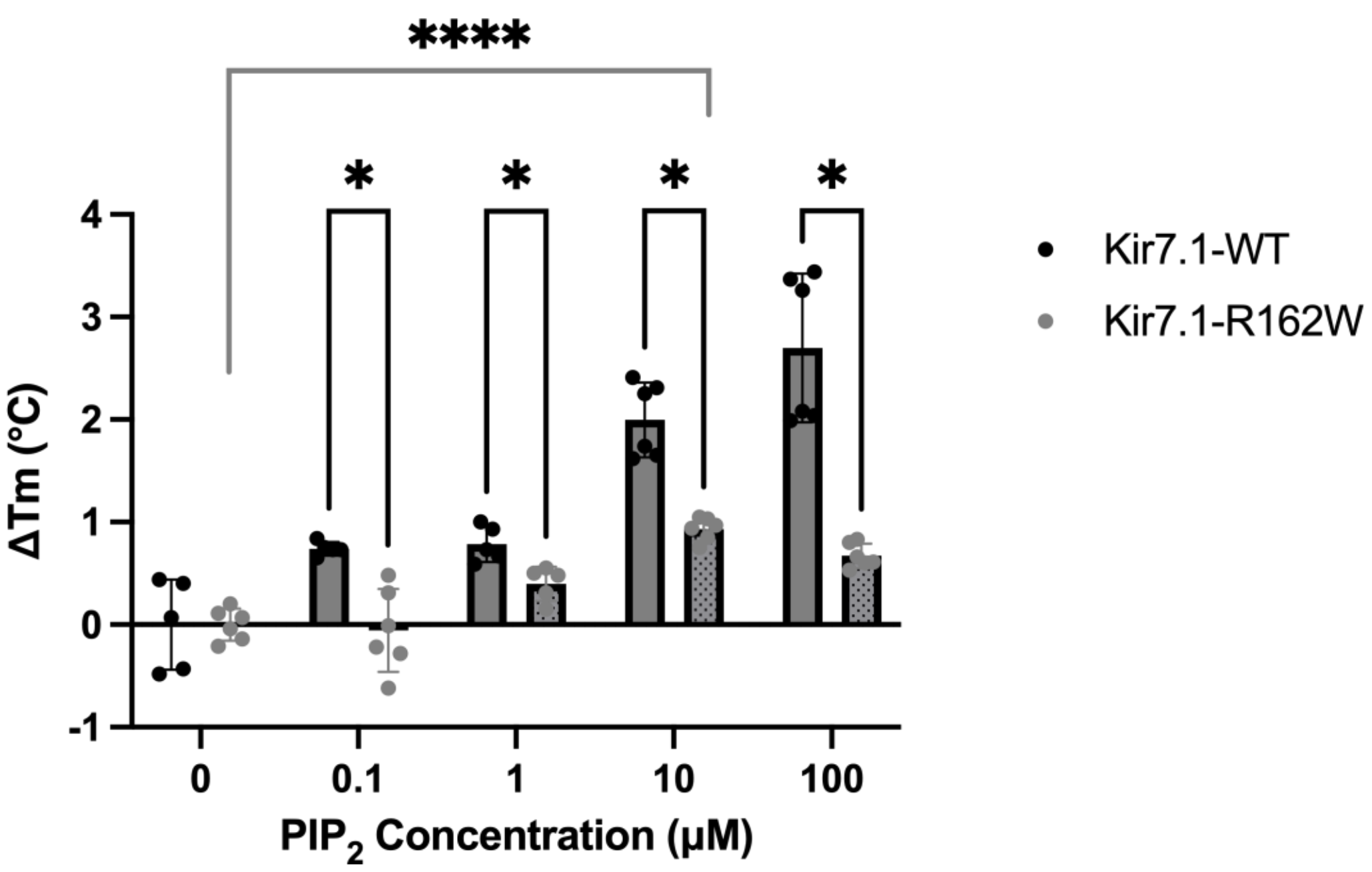

With these additions, the conclusions regarding the proposed pore-blocking mechanism of R162W would be much better supported, further strengthening the manuscript.

Therefore, I recommend further revision, as the additional control and the R162W concentration–response NanoDSF are important to substantiate the authors' claims.

Reviewer #3 (Remarks to the Author):

Summary:
The authors have revised their manuscript describing the cryo-EM structures of the human Kir7.1 channel in Apo, PIP2-bound, and R162W mutant forms. They propose a mechanism where the R162W mutation causes Snowflake Vitreoretinal Degeneration (SVD) through steric occlusion of the pore rather than loss of PIP2 affinity. Additionally, they propose that PIP2 binding opens the Helix Bundle Crossing (HBC) without docking the Cytoplasmic Domain (CTD), suggesting a novel activation intermediate distinct from the fully docked conformation reported in other Kir channels. They attribute this difference largely to the expression system and detergent conditions.
I have carefully reviewed the revised manuscript and the authors' responses to the

previous comments. I appreciate the authors' efforts in improving the clarity of the figures and rewriting sections to address the concerns regarding the lack of functional data. I also acknowledge the inclusion of NanoDSF statistical analysis and the citation of historical electrophysiological data to contextualize the work. However, while the analysis of the R162W mutant is structurally compelling and distinct from recent work in the field, the interpretation of the PIP2-bound wild-type structure remains a point of concern. The manuscript requires careful textual revision to avoid over-interpreting what is likely an experimentally derived conformational state as a definitive physiological "new active form".

Comments:

1. Interpretation of the PIP2-bound "Intermediate" State. In the revised manuscript and rebuttal, the authors argue that their structure (Mammalian cells/GDN) represents a more "native-like" or "intermediate" state compared to the Peisley et al. structure (Insect cells/DDM+CHS), which shows the canonical docked conformation. While I appreciate the authors' hypothesis, it is scientifically risky to claim the "extended" state is more physiological solely based on the expression system. In the broader context of Kir channel structural biology (e.g., Kir2.2, Kir3.2, Kir6.2), the docked conformation is widely accepted as the fully active state essential for channel gating.

It is equally plausible that the "extended" PIP2-bound state observed here represents a decoupled or desensitized state, possibly due to the loss of specific lipids during GDN purification that are required for the full CTD docking, rather than a functional "pre-activated" intermediate. The authors should balance their discussion further. Instead of implying the Peisley structure is less native due to CHS/Insect cells, the authors should explicitly acknowledge that their structure might capture a state where the CTD-TMD coupling is weak or uncoupled, and that the "docked" state observed by Peisley et al. represents the fully activated endpoint. The current text (Discussion) leans too heavily on the "intermediate" narrative without sufficient functional proof.

We thank the reviewer for their thoughtful comment and agree with the concern regarding overinterpretation. In response, we have revised the Discussion to provide a more balanced interpretation of the PIP2-bound structure. Specifically, we have removed language suggesting that the extended PIP2-bound conformation represents a definitive active state. Instead, we now discuss the possibility that this structure may represent a conformation with weakened TMD-CTD coupling due to reconstitution in GDN. We also acknowledge that the docked confirmation may represent the fully activated state.

2. The Mechanism of R162W vs. R162Q. I commend the authors for retaining the focus on the R162W mutation. This is the strongest point of differentiation from the R162Q structure. The mechanism of a steric block by the bulky Tryptophan side chain in the pore (R162W) is structurally distinct from the mechanism proposed for R162Q (which describe as stabilizing a docked but non-conductive state or altering gating kinetics). The R162Q mutation (Retinitis Pigmentosa) causes a gain-of-function, whereas R162W (SVD) causes a loss-of-function.

The authors should explicitly contrast their R162W structural mechanism with the R162Q mechanism described in the preprint in the Discussion section. Highlighting that different mutations at the same site can lead to disease via distinct structural

mechanisms (steric block vs. uncoupling/locking) would significantly add value to this publication and justify its coexistence with the other paper.

We thank the reviewer for this suggestion and agree that comparison with the R162Q mutation provides important context. We have therefore added discussion of the reported R162W structure and compare its structural and functional consequence with the R162W mutation described here.

3. Functional Validation Claims. In response to previous reviews, the authors declined to perform new electrophysiology, citing existing literature. While I accept that the basic function of Kir7.1 is established, the lack of functional data specific to the constructs and lipids used here creates a gap in correlating the structure to function. I appreciate that the authors have toned down the language regarding PIP2 "affinity" based on the NanoDSF data. The current conclusion that R162W does not abolish PIP2 interaction is reasonable. The authors should provide the text strictly adheres to "thermal stability" rather than "binding affinity".

We thank the reviewer for this clarification. In response, we have revised the manuscript to avoid describing the NanoDSF results in terms of PIP2 binding affinity. The text now used thermal stabilisation to describe the effect, which ensures that the interpretation of the NanoDSF data remains appropriately cautious and consistent.

4. Visualizing the Gating Cycle. The new schematic (Figure 5) helps summarize the authors' model. However, based on reviewer #1, the "Activated/Pre-activated" middle panel is potentially misleading if presented as the final conducting state. I suggest adding a visual note or question mark indicating that full activation (high conductance) likely requires the transition to the Docked state (as seen in other Kirs), to be consistent with the field's general consensus. This would make the model more robust and less susceptible to criticism that it ignores the canonical gating mechanism.

We thank the reviewer for this suggestion. We have now revised Figure 5 to avoid implying that the PIP2-bound extended conformation necessarily represents the final conducting state. The schematic now includes a question mark and an explanatory note. This modification provides a clearer representation of the uncertainties in the model and avoids overstating our interpretation.

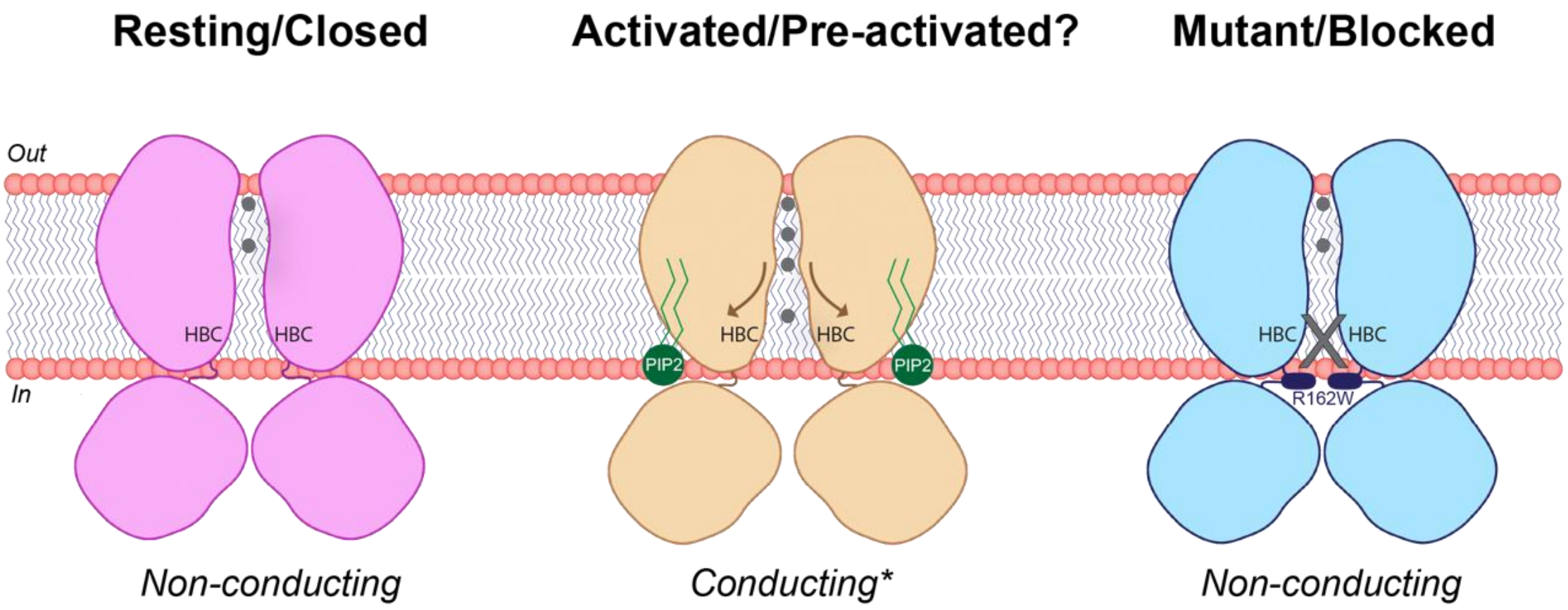

Figure 5. Schematic summary of Kir7.1 activation and pore blockage by R162W mutation. Left: Kir7.1 in the resting (closed) state in the absence of the activating lipid PIP_2. **Middle**: PIP_2-bound activated (or pre-activated) state with dilation at the helix bundle crossing. **Right**: The Snowflake Vitreoretinal Degeneration-associated R162W mutation introduces a steric block within the channel pore, preventing ion conductance. HBC: Helix bundle crossing. *Full activation may require further confirmational changes.

Dear Editor and Reviewers,

We sincerely thank the Editor and Reviewers for their careful evaluation of our revised manuscript. We are pleased that the revisions have been well received. We have addressed the remaining comment from the Editorial Board Member, which we believe improves the clarity of the manuscript.

How K^+ ions are coordinated in the conductance pore is fundamentally important in the channel field. If the authors model K^+ ions differently in these channel conformations, they need to explain it. I would suggest that they add some statements in the legend of Fig. 5 to avoid confusion.

We agree that clear description of K^+ coordination is essential. In response, we have revised the legend of Figure 5 to explicitly describe how potassium ions are coordinated in each conformational state, and to clarify that coordination within the selectivity filter is conserved across states.

The updated legend now reads (new text shown in red in the revised manuscript):

"Figure 5. Schematic summary of Kir7.1 activation and pore blockage by the R162W mutation. Left: Kir7.1 in the resting (closed) state in the absence of the activating lipid PIP_2. Potassium ions are coordinated by backbone carbonyl oxygens within the selectivity filter (G121, I120, and T119). Middle: PIP_2-bound activated (or pre-activated) state with dilation at the helix bundle crossing. Potassium ions remain coordinated by backbone carbonyl oxygens within the selectivity filter (T122, G121, and I120). Density corresponding to a fourth potassium ion was observed within the central cavity. Right: The Snowflake Vitreoretinal Degeneration-associated R162W mutation introduces a steric block within the channel pore, preventing ion conductance. Coordination of potassium ions within the selectivity filter is maintained by backbone carbonyl oxygens (G121, I120, and T119)."

We hope this clarification resolves the reviewer's concern.
