# Supplementary material for: Cryo-EM structure of the human Kir7.1 channel reveals the molecular basis of snowflake vitreoretinal degeneration disease
Source: Commun Biol. 2026 Jun 21;9:1311. doi: 10.1038/s42003-026-10436-0 (PMC13646276; doi:10.1038/s42003-026-10436-0)
Supplement: Supplementary file 2 [file 42003_2026_10436_MOESM2_ESM.pdf]

Supporting Information: Cryo-EM structure of the human Kir7.1 channel reveals the molecular basis of Snowflake Vitreoretinal Degeneration disease

Niamh O'Malley[1,4], Chady Nasrallah[2], Ashton Churchill[2], Jay Bertrand[2,3], Belinda R. Faust[2*], and B.A. Wallace[1*]

[1] Institute of Structural and Molecular Biology, Birkbeck University of London, Malet Street, London WC1E 7HX, UK.

[2] Vertex Pharmaceuticals, 86-88 Jubilee Ave, Milton, Abingdon OX14 4RW, UK.

[3] Present address: Evotec US, 303B College Rd E, Princeton, NJ 08540, USA.

[4] Present address: The Francis Crick Institute, 1 Midland Road, London NW1 1AT, UK.

*Address correspondence to B.A.W. b.wallace@bbk.ac.uk and B.R.F. belinda.faust@vrtx.com

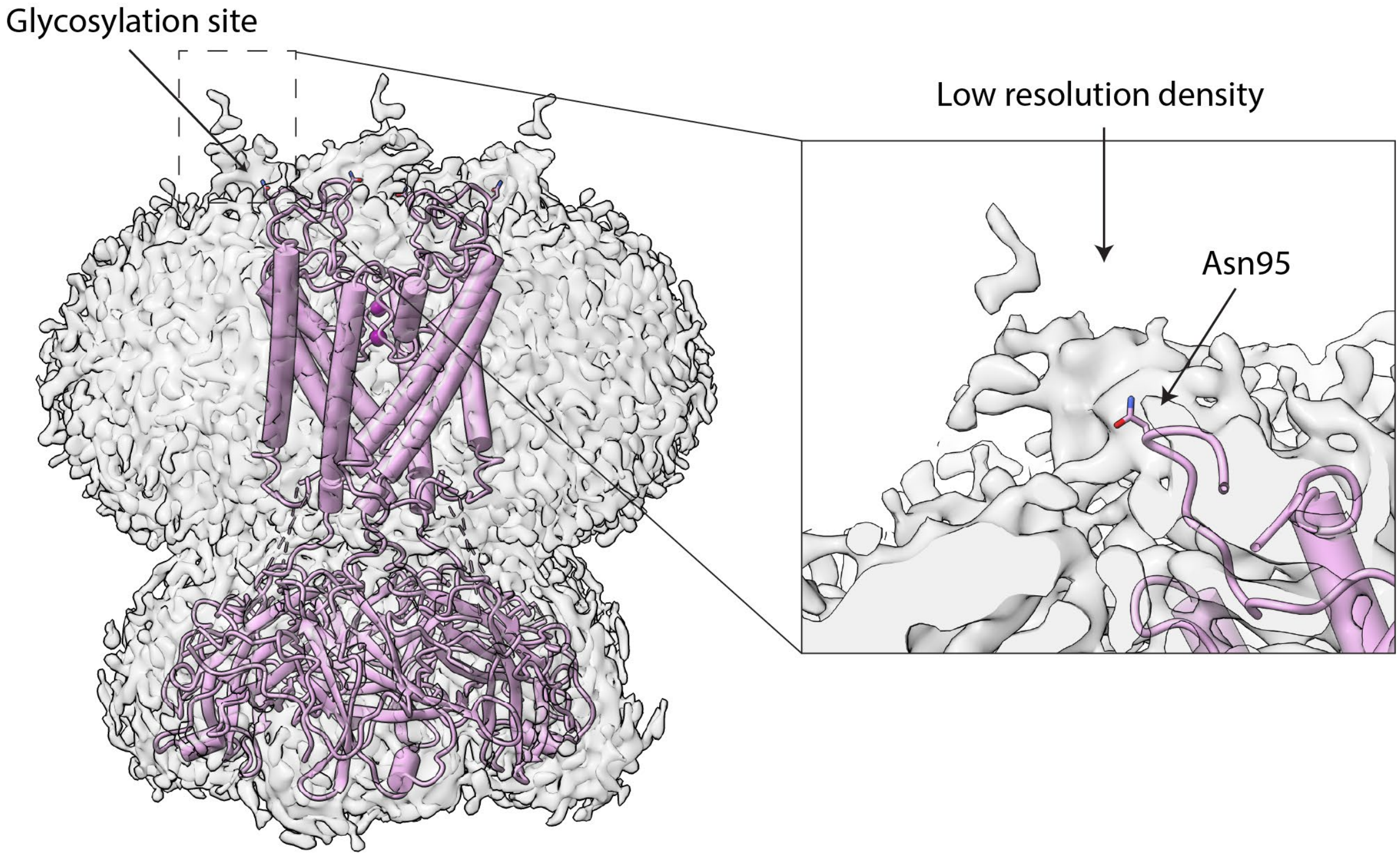

Supplementary Figure 1: EM map showing density representing glycosylated Kir7.1. Glycosylation site N95 within the extracellular turret in Kir7.1 showing low resolution density. Zoom in view shows density originating from N95.

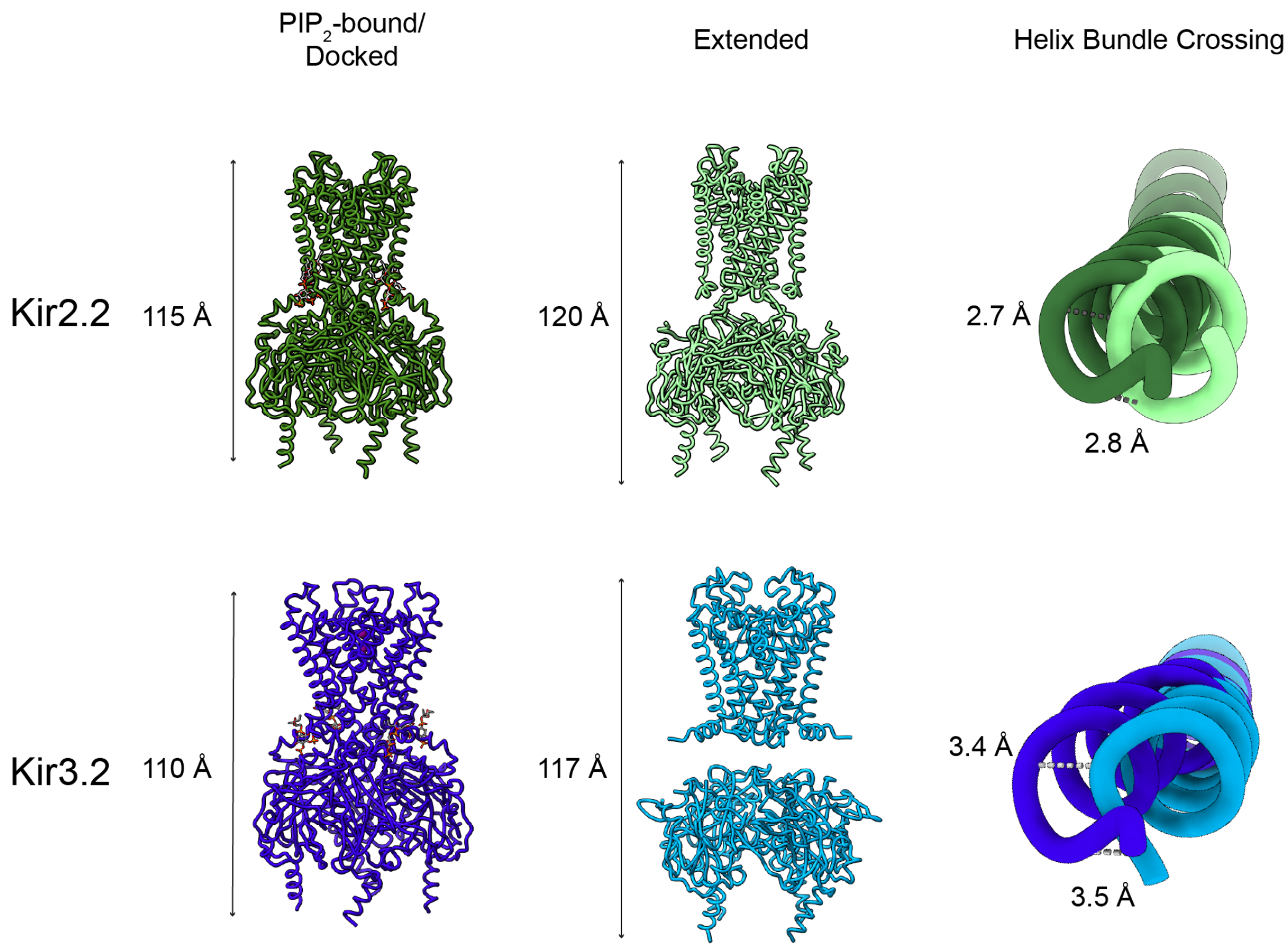

Supplementary Figure 2. Conformational differences between PIP_2-bound and extended states of Kir2.2 and Kir3.2 channels. Left: PIP_2-bound conformations of Kir2.2 and Kir3.2 show the cytoplasmic domain positioned closer to the membrane (Kir2.2: ~115 Å; Kir3.2: ~110 Å). **Middle:** In the extended conformations, the cytoplasmic domain disengages from the transmembrane domain, increasing the overall channel height (Kir2.2: ~120 Å; Kir3.2: ~117 Å). **Right:** Close-up view of the helix bundle crossing (HBC) from the intracellular side. In both channels, the inner transmembrane helix bends outwards around the conserved hinge glycine, resulting in a widened HBC and an expanded pore diameter. Distances were measured between the $C\alpha$ of corresponding residues using ChimeraX. Models used: Kir2.2 (PDB: 3SPI, 3JYC) and Kir3.2 (PDB: 6XIT, 6XIS).

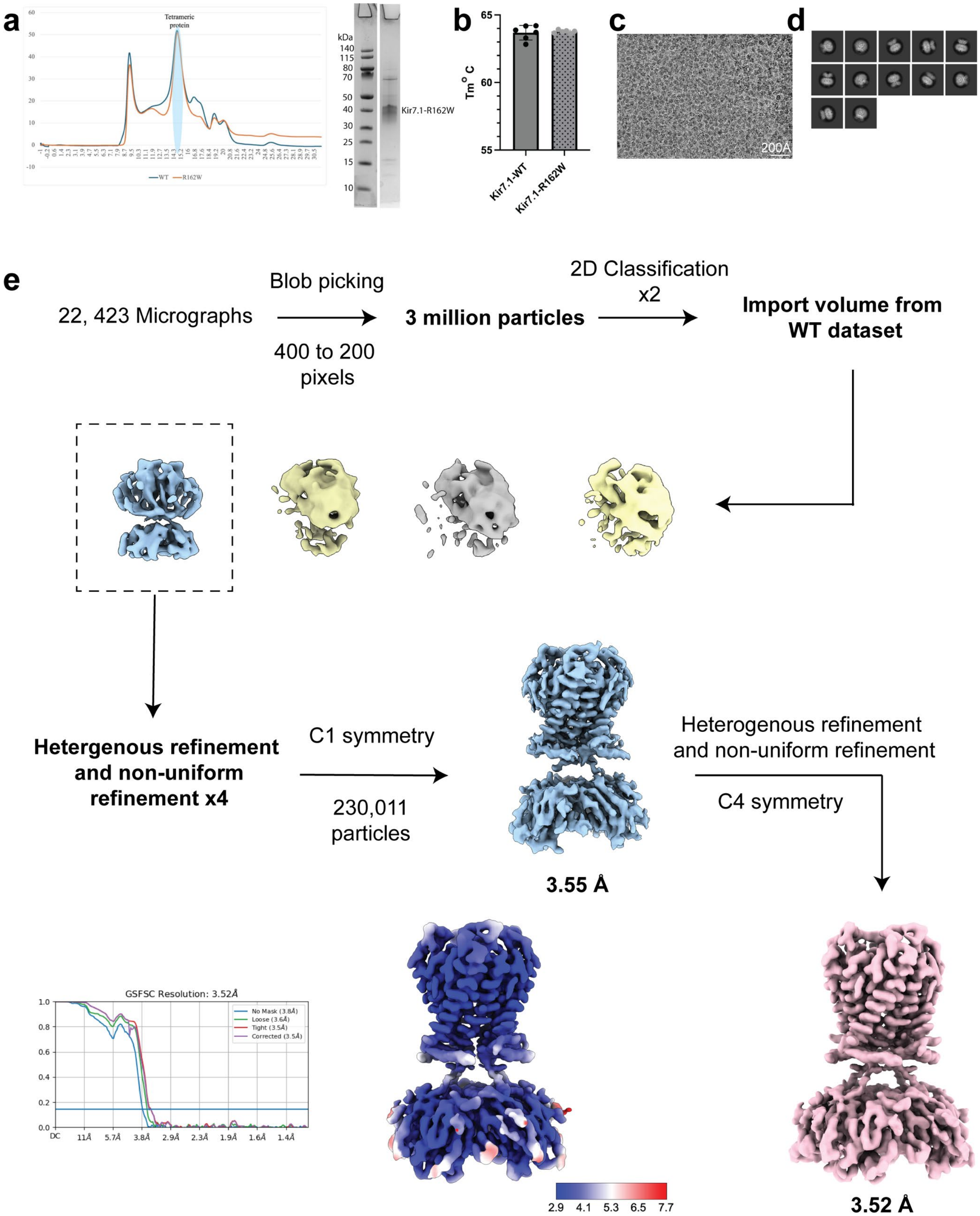

Supplementary Figure 3: Biochemical characterization and cryo-EM data processing workflow of Kir7.1-R162W. a Size exclusion chromatography trace of Kir7.1-R162W and SDS-PAGE of peak fraction. **b** Thermal unfolding measurements for Kir7.1-WT and Kir7.1-R162W. Error bars represent

the standard deviation calculated from six replicates across two independent experiments. **c** Representative micrograph of the purified Kir7.1-R162W. **d** 2D class averages. **e** Cryo-EM data processing workflow, with steps listed in bold and resolution listed below for each step in the refinement process.

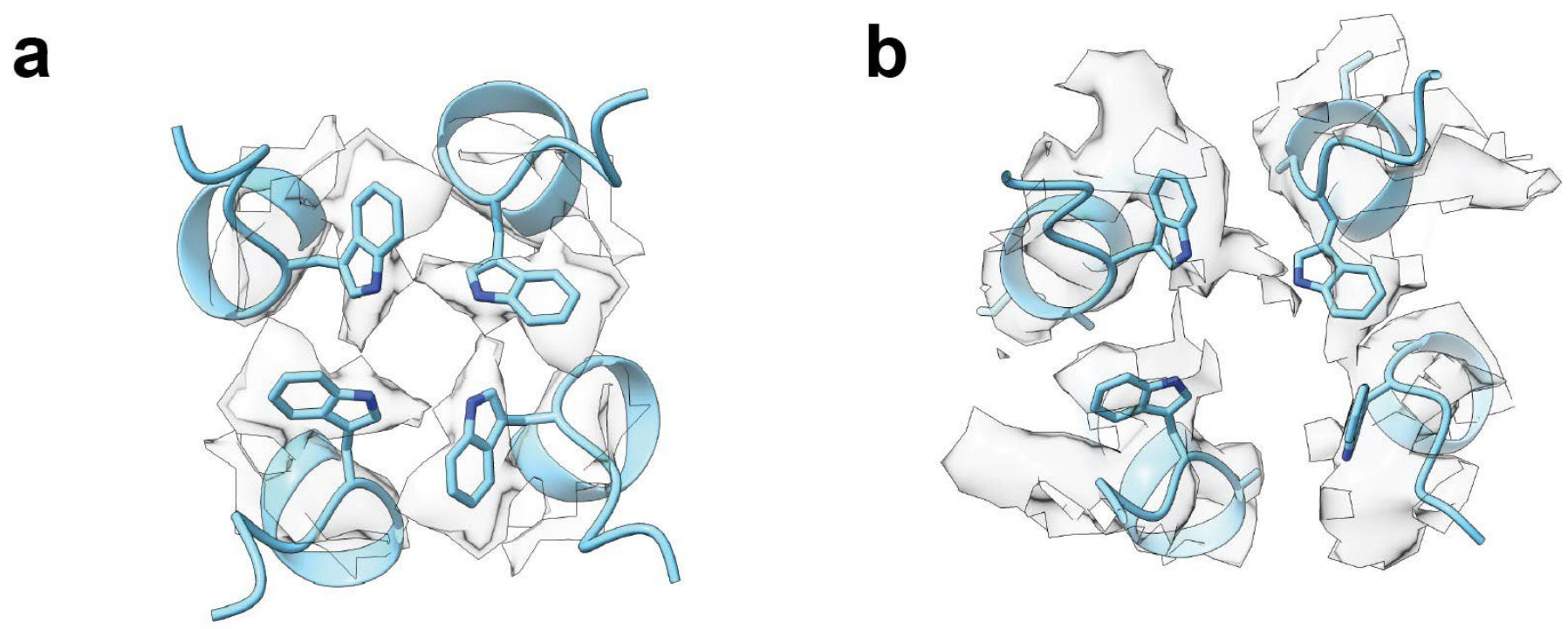

Supplementary Figure 4: Comparison of C1 and C4 symmetry enforcement for Kir7.1-R162W.
a Intracellular view of R162W sidechains within the EM density when processed using C4 symmetry.
b Intracellular view of R162W sidechains within the EM density when processed using C1 symmetry.

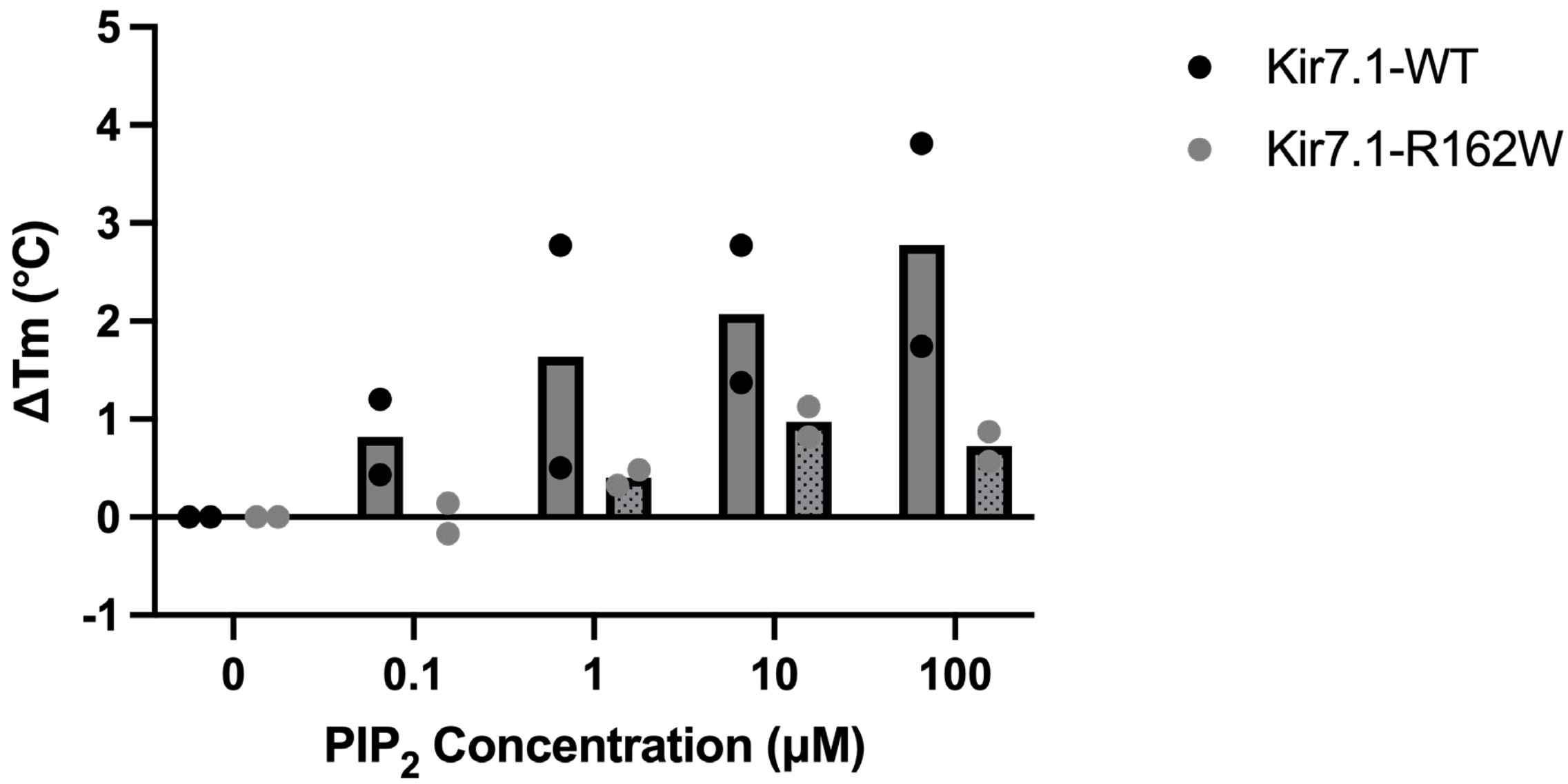

Supplementary Figure 5: Thermal stability of Kir7.1 WT and R162W mutant in response to increasing concentrations of PI(4,5)P_2 measured by NanoDSF. WT is shown in solid colour and R162W mutant in spots. Two biological replicates were performed, each including three technical replicates per condition. Data are represented as mean values per biological replicate.

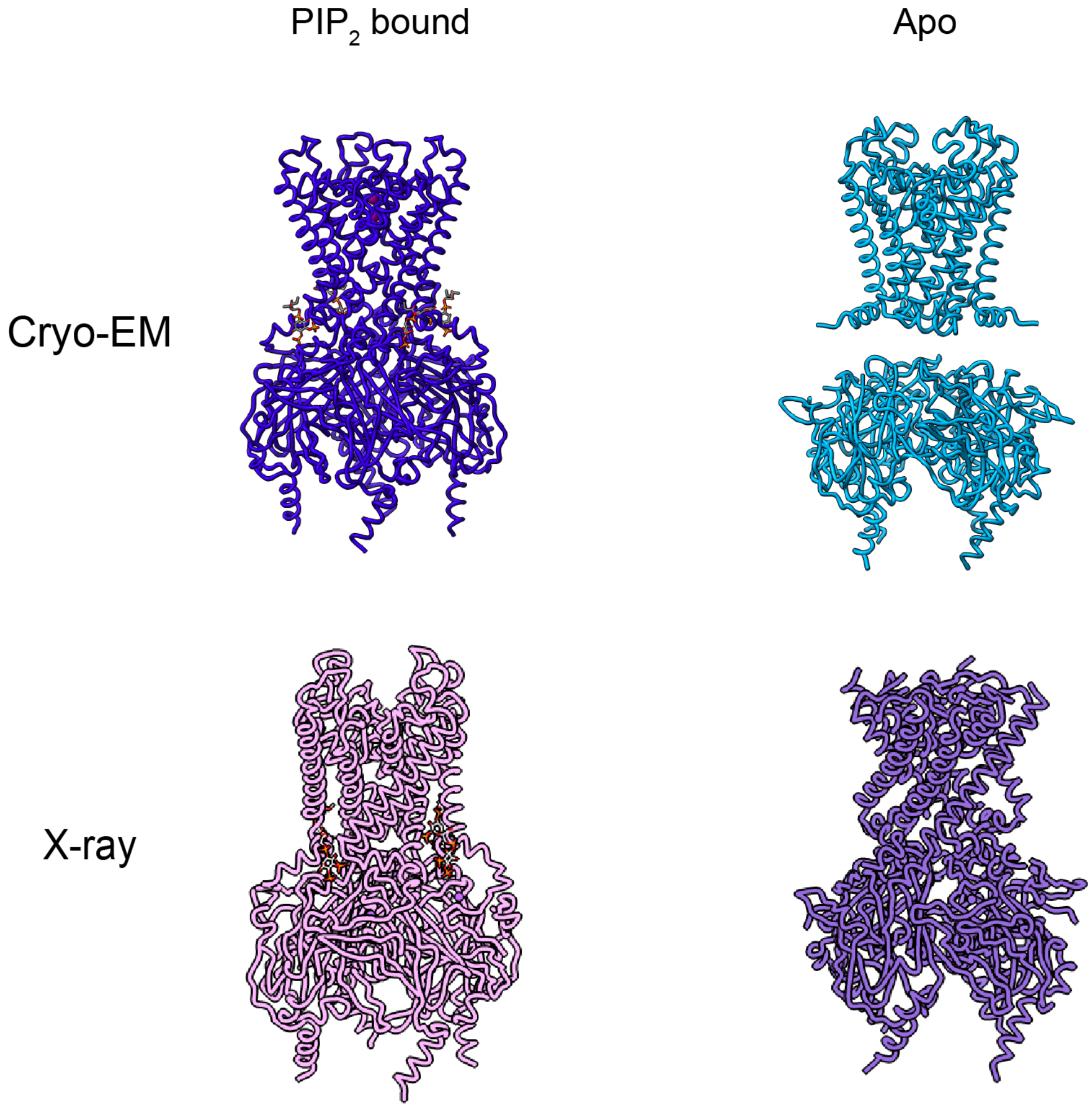

Supplementary Figure 6. Comparison of Kir3.2 Structures determined using Cryo-EM and X-ray crystallography in PIP$_2$-bound and apo states. Top: Cryo-EM structures of PIP$_2$-bound (6XIT) and Apo (6XIS) conformations of Kir3.2. **Bottom:** X-ray crystallographic structures of Kir3.2 in PIP$_2$-bound (3SYA) and Apo forms (3SYO)

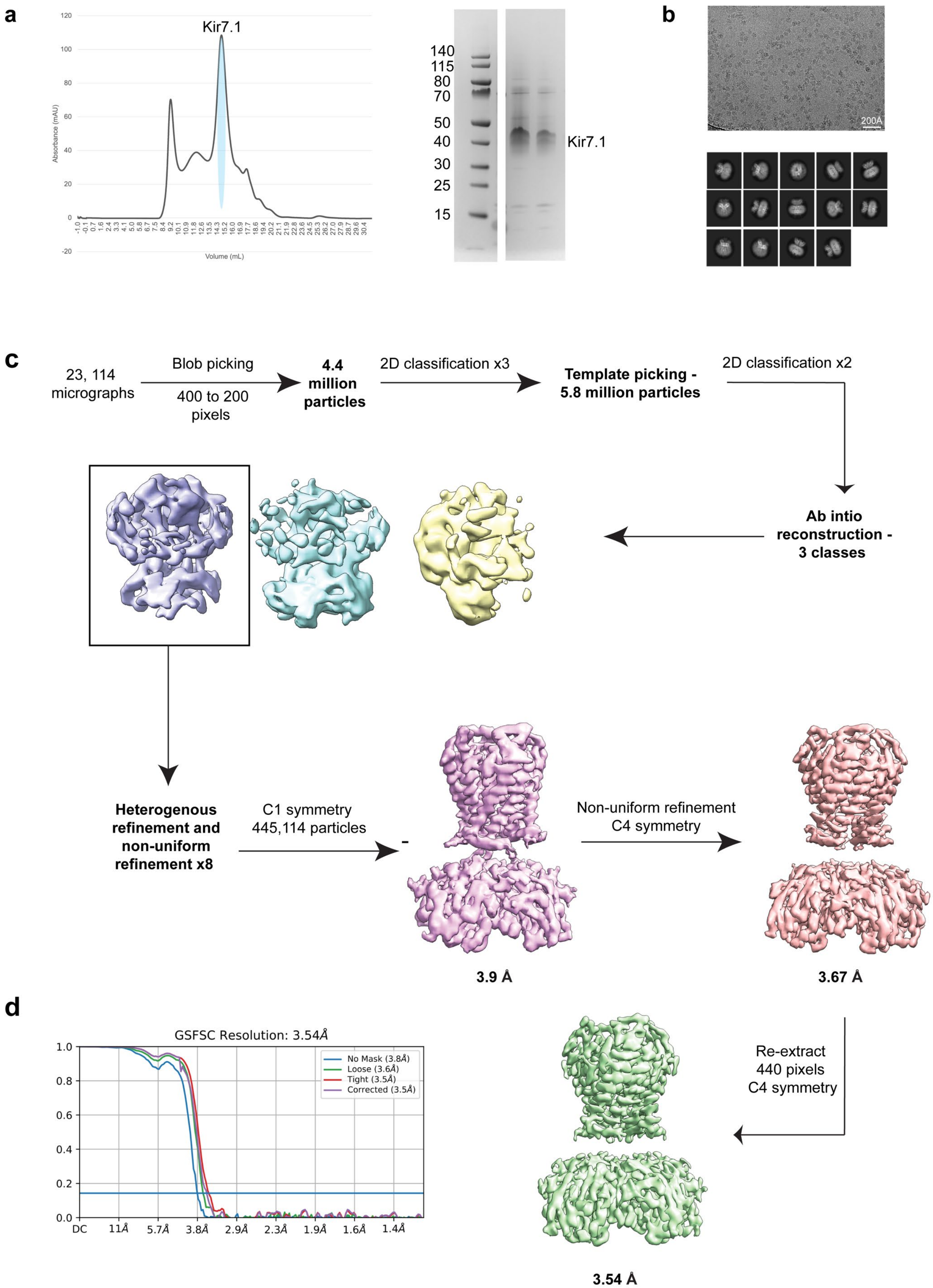
a
Kir7.1
Absorbance (mAU)
Volume (mL)
140
115
80
70
50
40
30
25
15
Kir7.1
b
200Å
c
23, 114 micrographs
Blob picking
400 to 200 pixels
4.4 million particles
2D classification x3
Template picking - 5.8 million particles
2D classification x2
Ab intio reconstruction - 3 classes
Heterogenous refinement and non-uniform refinement x8
C1 symmetry 445,114 particles
3.9 Å
Non-uniform refinement C4 symmetry
3.67 Å
Re-extract 440 pixels C4 symmetry
3.54 Å
d
GSFSC Resolution: 3.54Å
No Mask (3.8Å)
Loose (3.6Å)
Tight (3.5Å)
Corrected (3.5Å)
DC
11Å
5.7Å
3.8Å
2.9Å
2.3Å
1.9Å
1.6Å
1.4Å

Supplementary Figure 7: Biochemical characterization and cryo-EM data processing workflow of Apo-WT-Kir7.1. a (Top left) Size exclusion chromatography trace of Kir7.1. (Top middle) Protein samples of the indicated Kir7.1 fractions were subjected to SDS-PAGE and stained with Coomassie-blue**. b** (Top) Representative micrograph of the purified Apo-WT-Kir7.1 and (bottom) 2D class averages. **c** Cryo-EM data processing workflow, with steps listed in bold and resolution listed below for each step in the refinement process. **d** GSFSC curve for final refinement.

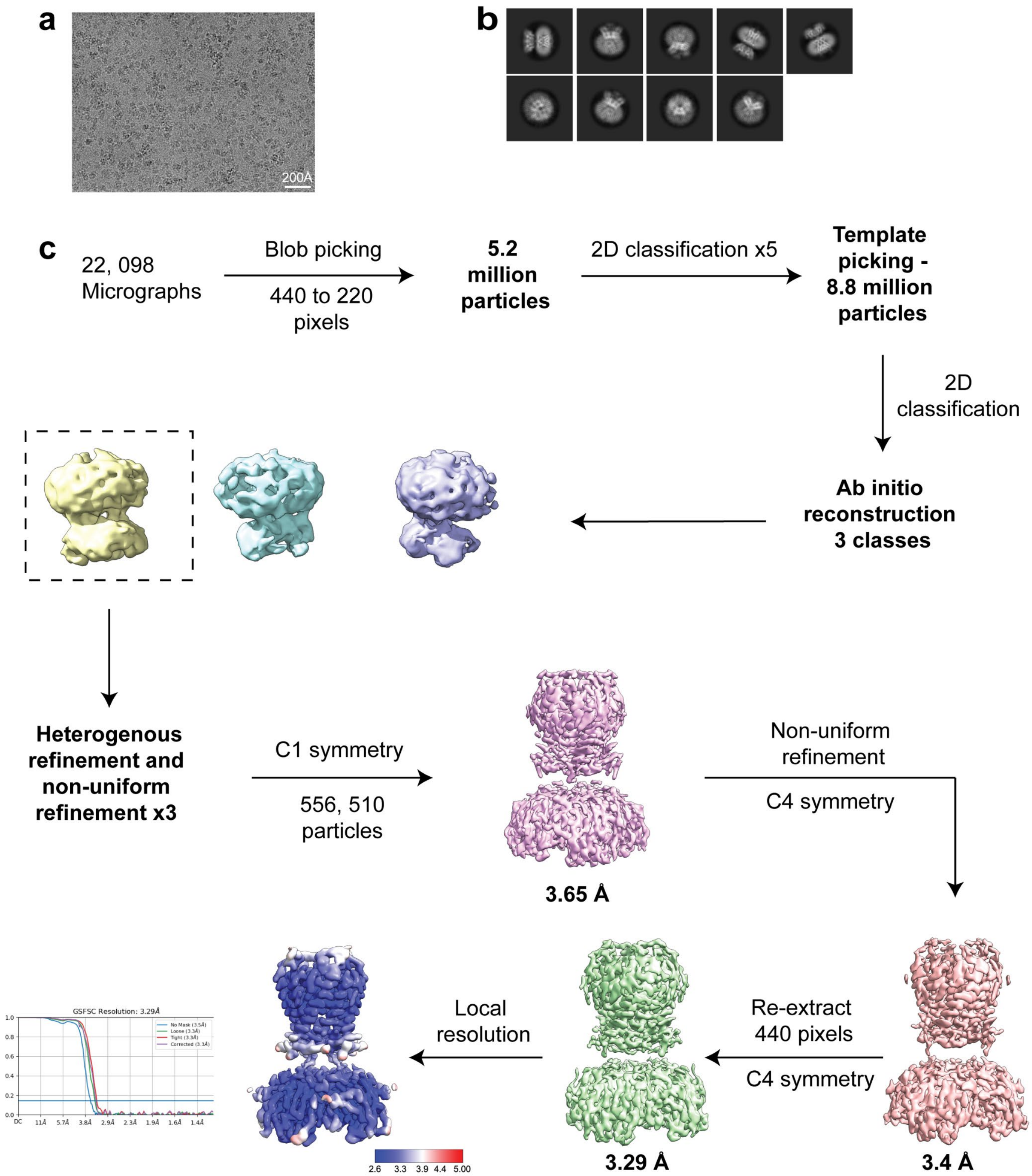

Supplementary Figure 8: Cryo-EM data processing workflow of WT-Kir7.1-PIP$_2$. a Representative micrograph of the purified Kir7.1-PIP$_2$. **b** 2D class averages. **d** Cryo-EM data processing workflow, with steps listed in bold and resolution listed below for each step in the refinement process.

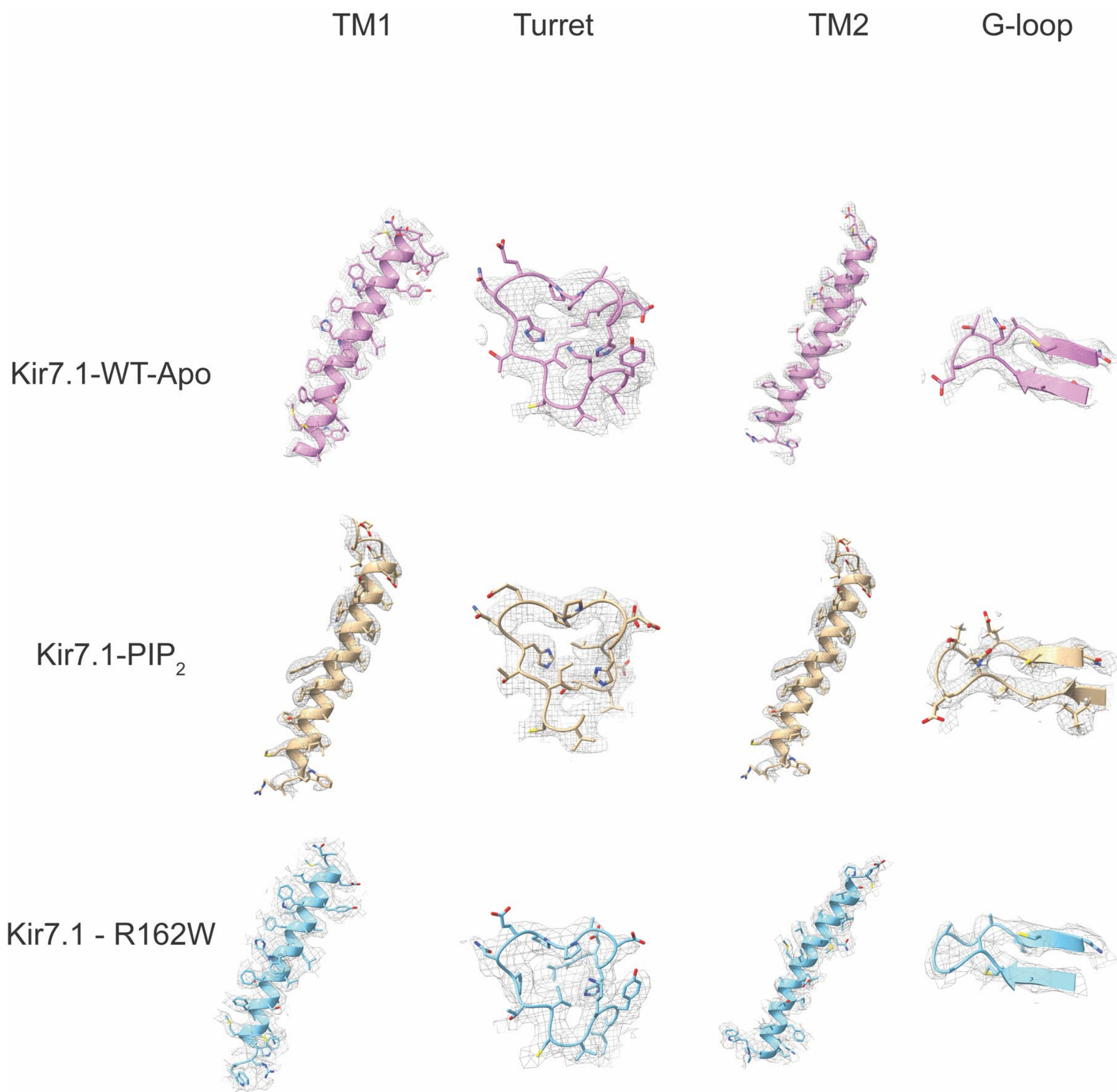

Supplementary Figure 9: Densities for WT, PIP$_2$-bound and R162W mutant structures. Local densities, shown as grey meshes, were prepared in ChimeraX at 3.35-3.55 σ contour level.

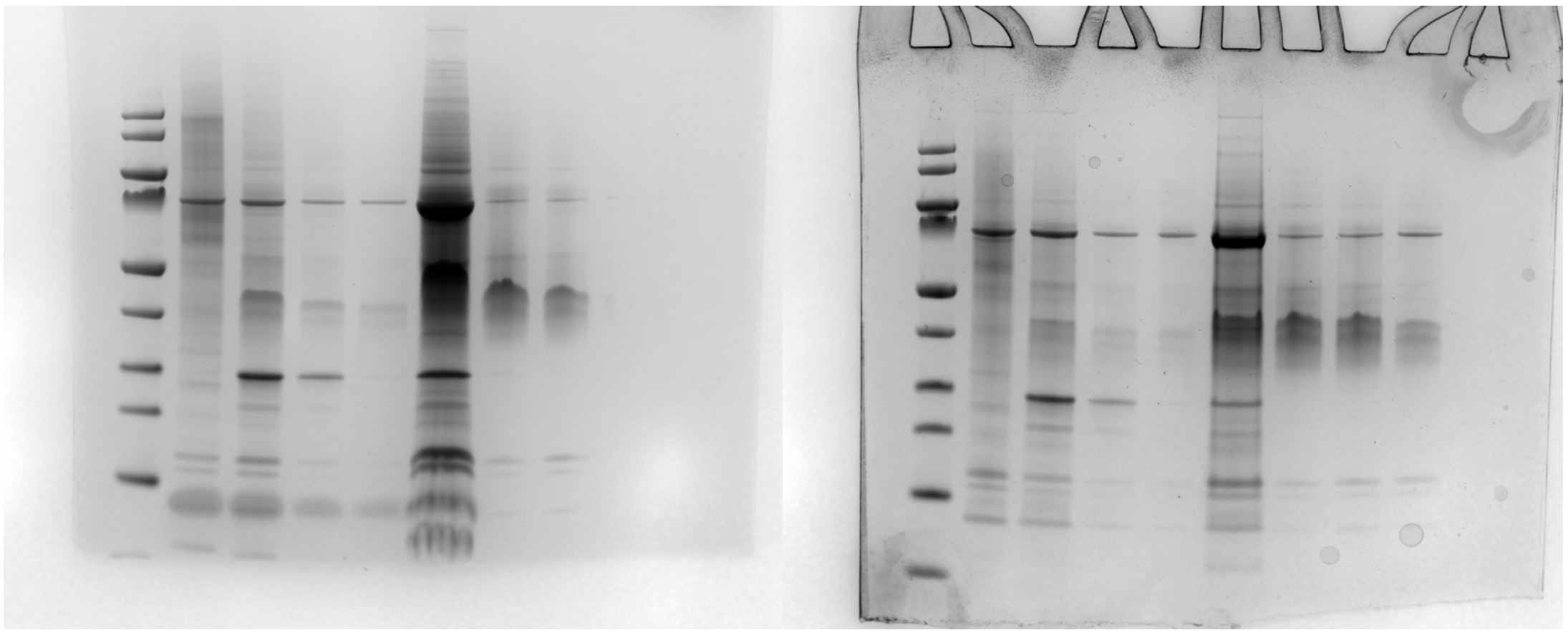

Supplementary Figure 10: Uncropped images of SDS-PAGE. Left: Original uncropped gel image corresponding the cropped regions in Supplementary Figure 7. **Right:** Original uncropped gel image corresponding the cropped regions in Supplementary Figure 3.
