# Supplementary material for: Cryo-EM structure of the human Kir7.1 channel reveals the molecular basis of snowflake vitreoretinal degeneration disease
Source: Commun Biol. 2026 Jun 21;9:1311. doi: 10.1038/s42003-026-10436-0 (PMC13646276; doi:10.1038/s42003-026-10436-0)
Supplement: Supplementary file 4 [file 42003_2026_10436_MOESM3_ESM.docx]

**Description of Additional Supplementary File**

File name: Supplementary Data
Description: The data behind the graphs in the paper
